# Molecular inotropy mediated by cardiac miR-based PDE4D/PRKAR1α/phosphoprotein signaling

**DOI:** 10.1038/srep36803

**Published:** 2016-11-11

**Authors:** Fikru B. Bedada, Joshua J. Martindale, Erik Arden, Joseph M. Metzger

**Affiliations:** 1 Department of Integrative Biology and Physiology, University of Minnesota Medical School, 6-125 Jackson Hall, 321 Church Street SE, Minneapolis, MN 55455 USA

## Abstract

Molecular inotropy refers to cardiac contractility that can be modified to affect overall heart pump performance. Here we show evidence of a new molecular pathway for positive inotropy by a cardiac-restricted microRNA (miR). We report enhanced cardiac myocyte performance by acute titration of cardiac myosin-embedded miR-208a. The observed positive effect was independent of host gene myosin effects with evidence of negative regulation of cAMP-specific 3′,5′-cyclic phosphodiesterase 4D (PDE4D) and the regulatory subunit of PKA (PRKAR1α) content culminating in PKA-site dependent phosphorylation of cardiac troponin I (cTnI) and phospholamban (PLN). Further, acute inhibition of miR-208a in adult myocytes *in vitro* increased PDE4D expression causing reduced isoproterenol-mediated phosphorylation of cTnI and PLN. Next, rAAV-mediated miR-208a gene delivery enhanced heart contractility and relaxation parameters *in vivo*. Finally, acute inducible increases in cardiac miR-208a *in vivo* reduced PDE4D and PRKAR1α, with evidence of increased content of several complementary miRs harboring the PDE4D recognition sequence. Physiologically, this resulted in significant cardiac cTnI and PLN phosphorylation and improved heart performance *in vivo*. As phosphorylation of cTnI and PLN is critical to myocyte function, titration of miR-208a represents a potential new mechanism to enhance myocardial performance via the PDE4D/PRKAR1α/PKA phosphoprotein signaling pathway.

Myocardial performance is tightly linked to the contractile state of the cardiac myocyte^1^. Inotropic drugs that target myocyte contractility have been in clinical practice for decades; however, these have noted limitations^2^,^3^. Accordingly, there is an urgent need to discover new inotropic agents with unique mechanisms of action for enhancing cardiac contractility^4^. MicroRNAs (miRs), which are highly conserved non-coding small RNAs that regulate gene expression^5^,^6^,^7^, are known to affect heart development and growth^8^,^9^,^10^,^11^,^12^ and are altered in cardiac disease^13^,^14^,^15^,^16^,^17^. However, little is known whether a direct or indirect pathway exists between miRs and their targets influencing cardiac myocyte performance. Recently, down-regulation of miR-208a was shown to cause cardiac defects in *Trbp*cKO hearts and transgenic overexpression of miR-208a could rescue cardiac function through suppression of *Sox6* in Trbp knockout mice^16^. Interestingly, abnormal sarcomere organization and decreased phosphorylation of the regulatory myosin light chain-2 (MLC2), a critical cytoskeletal regulator, has been observed in miR-1 null cardiac myocytes suggesting that miR-1 is required for postnatal cardiac function^18^. Further, miRs have been identified that negatively regulate myocyte contractility^19^, raising the possibility that other miRs might be capable of enhancing myocyte function through direct or indirect effects.

Cardiac myosin isoforms are central to cardiac contractility^20^. Down-regulation of the fast *MYH6* isoform is linked to failing human heart performance^21^ and direct *MYH6* gene transfer boosts failing myocyte contractility^22^. The *MYH6* gene embeds the cardiac restricted miR-208a whose expression, like the host gene, can be depressed in chronic heart disease^16^,^17^,^23^,^24^. It is unclear, however, whether cardiac specific miR-208a has a direct or indirect effect on cardiac function independent of host gene myosin effects. In humans, miR-208a is significantly down-regulated in patients with atrial fibrillation (AF)^17^ and with chronic cardiomyopathy^24^. In rodents, chronic miR-208a deficiency is reported to cause heart performance deficits^25^,^26^ and chronic transgenic overexpression of miR-208a induces arrhythmias in the absence of sarcomere structural alterations^25^. On the other hand, beneficial effects of miR-208a have been reported in miR-208 mutant animals showing virtually no hypertrophy of cardiac myocytes or fibrosis in response to pressure overload induced by thoracic aortic banding (TAB)^26^. Furthermore, therapeutic inhibition of miR-208a improves cardiac function and survival during heart failure^27^. These findings suggest that titrating miR-208a content is critical to overall cardiac function^16^,^25^,^26^; however, a mechanistic understanding of this effect is currently unresolved.

We report here that acute increases in miR-208a expression can directly enhance myocyte performance. We find that the kinetics of these effects are too rapid to be caused by host gene myosin isoform switching. Subsequent informatics and experimental results revealed a new mechanism whereby miR-208a has a direct effect to suppress cAMP-specific 3′, 5′-cyclic phosphodiesterase 4D (PDE4D) and this is amplified through an indirect effect on regulatory subunit of PKA (PRKAR1α), a critical enzyme for myocyte inotropic signaling. Mechanistically, we report that miR-208a-mediated decreased PDE4D and PRKAR1α content leads to increased PKA-site specific phosphorylation of vital cardiac phosphoproteins troponin I and phospholamban. These cardiac phosphosubstrates are well known modulators of myocyte performance^28^,^29^,^30^ and are sufficient to account for the observed improved cellular performance. Furthermore, miR-208a regulates additional cardiac expressed miRs harboring the PDE4D recognition site, suggesting a concerted effect with other miRs. These findings are evidence of a new mechanism for miRs by acutely enhancing myocyte performance via the PDE4D/PRKAR1α/PKA/myofilament signaling pathway.

## Results

### MiR-208a enhances myocyte performance independent of host gene myosin effects

**Figure 1:**
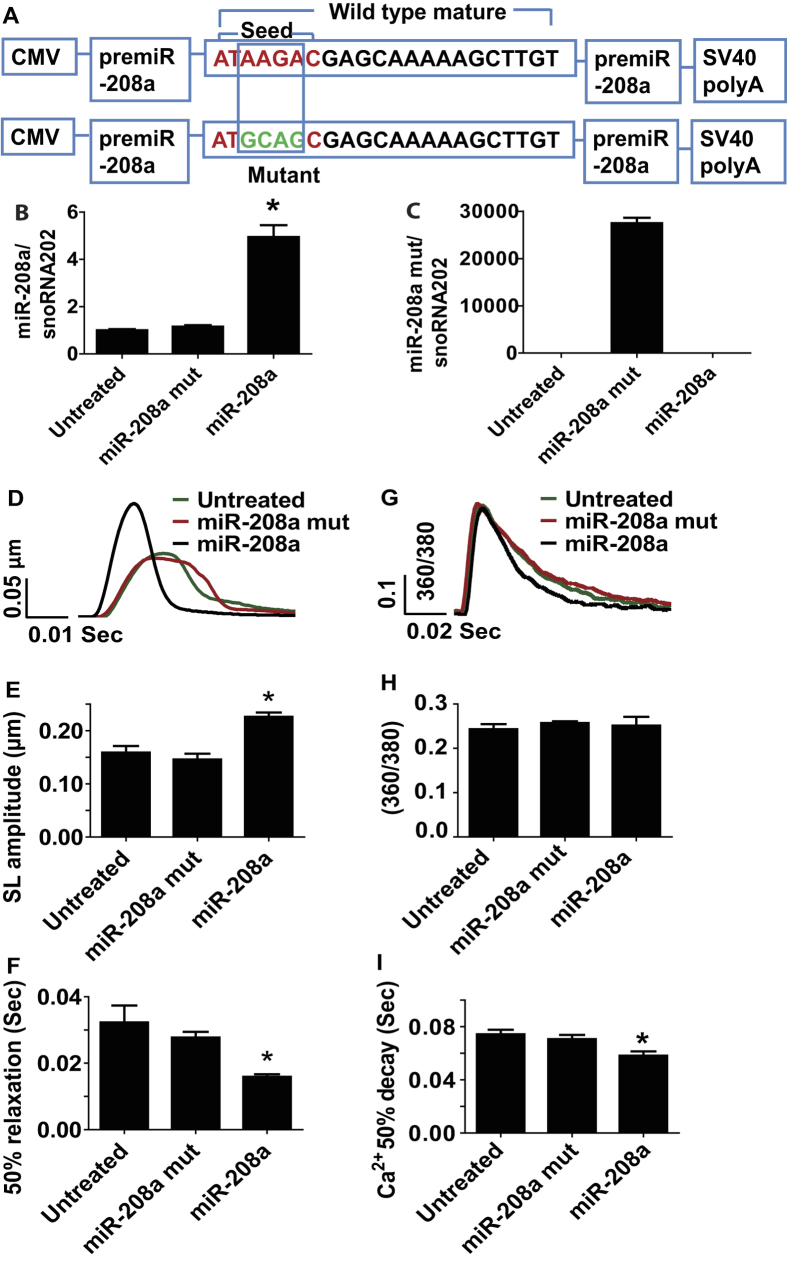
MiR-208a confers positive inotropy and lusitropy to adult ventricular myocytes. Constructs used for the generation of adenovirus expressing miR-208a (AdmiR-208a) and miR-208a mutant in adult myocytes (**A**). Expression of miR-208a and miR-208a mutant in cardiac myocytes (**B,C**). Superimposed representative SL shortening traces collected from field stimulated (0.5 Hz) untreated control (green), miR-208a mutant (red) and miR-208a-transduced (black) cardiac myocytes after gene transfer (**D**). Summaries of cardiac contraction and relaxation parameters, including SL shortening amplitude (**E**) and time to base line 50% (**F**). Data are shown as the means+/− SEM n = 27–31 myocytes. *P < 0.05 by One-way ANOVA. Representative calcium traces collected from field stimulated (0.5 Hz) untreated control (green), miR-208a mutant (red) and miR-208a-transduced (black) myocytes after gene transfer (**G**). Summaries of calcium amplitude and calcium decay parameters, including calcium amplitude (**H**) and 50% decay rate (**I**). Data are shown as mean +/− SEM, n = 9–11 myocytes. *P < 0.05 by One-way ANOVA.

To test for a possible direct effect of miR-208a on adult ventricular myocyte contractility, we generated adenovirus expressing miR-208a (mouse sequence; AdmiR-208a) and transduced adult rat ventricular cardiac myocytes in serum-free primary culture conditions. As a control, we generated miR-208a mutant by altering four nucleotides from the seed sequence of wild type miR-208a (Fig. 1A). At 48 hours post-transduction of freshly isolated adult myocytes with miR-208a, there was a 4-fold increased expression of miR-208a compared to myocytes harboring miR-208a mutant or non-transduced controls (Fig. 1B). The mutant is not detected in myocytes harboring wild type miR-208a or in non-transduced controls (Fig. 1C). Figure 1D shows representative single adult cardiac myocyte shortening traces collected from untreated control (green), miR-208a mutant (red) and miR-208a-transduced (black) myocytes.

Myocytes transduced with miR-208a had increased sarcomere length (SL) shortening amplitude (myocyte positive inotropy) compared to controls and to miR-208a mutant treated myocytes P < 0.05 (Fig. 1E), with no change in resting SL. In addition, miR-208a transduced myocytes had significantly faster relaxation (positive lusitropy), as assessed by a significant decrease in time to bl 50% compared to miR-208a mutant and untreated control myocytes (Fig. 1F). Figure 1G shows representative Ca^2+^ transient records collected from a field-stimulated untreated control (green), miR-208a mutant (red) and miR-208a-transduced (black) myocytes on day two after gene transfer. MiR-208a transduced myocytes had no change in Ca^2+^ amplitude (Fig. 1H) or resting Ca^2+^. MiR-208a transduced myocytes showed faster Ca^2+^ decay as determined by an increase in 50% Ca^2+^ decay rate compared to miR-208a mutant, and untreated control (Fig. 1I) P < 0.05. MiR-208a transduced myocytes had no change in αMHC/βMHC isoform composition (Figure S1G,H). MiR-208a transduced myocytes had normal sarcomeric actinin expression and structure and normal rod shaped morphology and size (Figure S1A,F,I).

To further determine the direct effect of miR-208a gene transfer on contraction as a function of frequency stimulation (Figure S2), myocytes were subjected to different bouts of frequency stimulation: 0.5, 1, 2 and 4 Hz as depicted by sarcomeric length (SL) traces for untreated (A), miR-208a mutant (C) and miR-208a (E). As shown in the right panels, 4 Hz stimulation data for non-transduced control (B) miR-208a mutant (D) were depressed and had episodes of irregular contractile responses compared to miR-208a transduced myocytes (F) which were highly responsive to cellular-based cardiac stress testing. Further, change in sarcomere length percent shortening as a function of frequency (Figure S2G) indicated that miR-208a transduced myocytes have stronger contractions compared with miR-208a mutant and non-transduced controls P < 0.05. Overall, miR-208a transduced myocytes had increased contractility and were highly responsive after stimulation at 0.5, 1, 2 and 4 Hz, whereas control myocytes were unable to respond at the higher stimulation rates (Figure S2B,D,F).

### MiR-208a promotes enhanced cardiac performance via suppression of PDE4D

**Figure 2:**
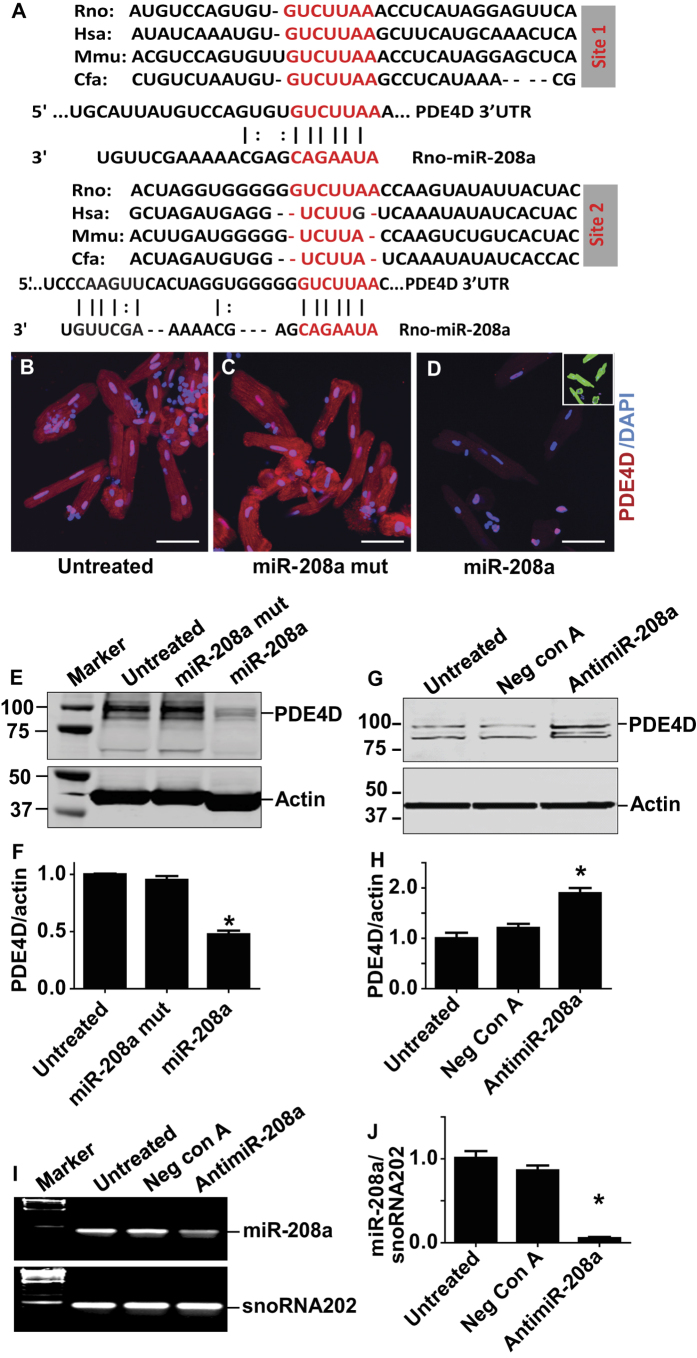
MiR-208a suppresses PDE4D in adult ventricular myocytes. (**A**) Two sites of PDE4D 3′ UTR sequence alignment are shown across species highlighting the seed match sequences of PDE4D 3′UTR (red) complementary to the seed sequences of miR-208a. Expression of PDE4D as assessed by immunohistochemistry (**B–D**). Significant reduction of total PDE4D staining was observed using a PDE4D specific antibody both in the cytoplasm and nucleus of miR-208a transduced but not in control myocytes **(B–D**). Sarcomeric actinin (green) shows the presence of the myocytes that were also stained by PDE4D (**D**; small box green for actinin). The images were taken with 40x objective confocal microscopy and bar shows scale of 50 μm. Western blots show that PDE4D was reduced significantly in miR-208a transduced but not in untreated or miR-208a mutant myocytes (**E,F**). Effects of pharmacologic inhibition of miR-208a via miR-208a power inhibitor that increased PDE4D content (**G,H**). Note that the Western blot in **G** was run longer resulting in distinct band separation versus **E**. Acute suppression of miR-208a in adult ventricular cardiac myocytes by antimiR-208a (**I,J**). Data are shown as mean +/− SEM, n = 3 *P < 0.05.

To gain molecular insight into the mechanism underlying the enhanced function of miR-208a transduced myocytes, we analyzed in silico for putative miR-208a targets, focusing on potential inotropic signaling pathways. From the TargetScan database, versions 3.1 and 4.1 (http://www.targetscan.org/), cAMP-specific 3′,5′-cyclic phosphodiesterase 4D (PDE4D) (but not other PDEs) were indicated as a potential candidate for miR-208a at two sites (Fig. 2A), although the second site is less well conserved. Computational approaches have been widely used by several groups previously^23^,^25^,^26^. For instance, Callis *et al*.^25^ utilized the Web-based Targetscan Human database to select predicted miR-208a target genes. These miR-208a targets include myostatin (GDF8), GATA4 and THRAP1^25^. Similar to our approach, Ikeda *et al*.^23^ used computational algorithms to find candidate genes as targets of miR-1 that mediate cardiac myocyte hypertrophy. Further, van Rooij *et al*.^26^ also used a computational approach to experimentally validate THRAP1 as miR-208a target. We have also used THRAP1 as positive control to experimentally validate the effect of miR-208a (Figure S9). Next, as a unbiased approach for PDE4D, we evaluated specific effects of miR-208a focusing on several cAMP hydrolyzing phosphodiesterase (PDEs) families in isolated myocytes by quantitative RT-PCR (Figure S3). Data show that miR-208a has no effect on the PDE4A or PDE4B consistent with lack of seed match sequence (Figure S3A,B). The fourth subtype of PDE4, PDE4C was not investigated because it is not expressed in the heart^31^. Consistent with the presence of a seed match sequence, significant reduction of PDE4D isoforms PDE4D3 and PDE4D9 were observed in miR-208a treated myocytes (Figure S3D,E). Further, with the exception of PDE8A, no significant effect of miR-208a on other cAMP hydrolyzing PDEs, including PDE1A, PDE3A and PDE8B, was observed (Figure S3F–I).

After elucidating the specificity of miR-208a for PDE4D subtypes, we next focused on expression and localization of PDE4D protein by immunohistochemistry using confocal image analysis (Fig. 2B–D). Significant reduction of total PDE4D staining was observed by a PDE4D specific antibody, both in the cytoplasm and nucleus of miR-208a transduced, but not in mutant or control myocytes (Fig. 2B–D). To help visualize the presence of myocytes in miR-208a treated cells, DAPI/α-actinin staining was used (small box green for actinin; Fig. 2D). Western blot analysis from a previous study demonstrated the presence of PDE4B and PDE4D subtypes in the nuclei as well as enrichment in nuclear envelope^32^. Further, inhibition of PDE4 or ablation of the PDE4D gene in mice prolonged cytoplasmic PKA activation and enhanced nuclear PKA responses^33^. Thus, our observation is consistent with the fact that PDE4D could be a nuclear enriched PDE4 subtype that also controls cAMP/PKA signaling in the nucleus^32^,^33^. The extent of expression of PDE4D was assessed in individual myocytes at low optical magnification (20X). Representative immunofluorescence images are shown for untreated, miR-208a mutant and for miR-208a (Figure S4A–D). To help visualize the presence of myocytes in miR-208a treated cells DAPI staining was used (Figure S4D). The time course analysis of suppression of PDE4D post-miR-208a gene delivery in individual myocytes indicated marked suppression of PDE4D by miR-208a at day two (Figure S5A–I). This is in keeping with the reported short half-life of PDE4D (4hrs)^34^. Western blot data confirmed that miR-208a has a direct effect to negatively regulate expression of PDE4D (Fig. 2E,F). In addition, pharmacologic inhibition of miR-208a increased PDE4D content (Fig. 2G,H). Longer run blots showed the other PDE4D isoform bands are well separated (Fig. 2G vs. 2E). Significant reduction in miR208a was obtained by delivery of antimiR-208a (Fig. 2I,J). We verified pharmacologic inhibition of miR-208a using TaqMan microRNA expression assay system (Applied Biosystems). In comparison, PDE5A expression was not affected by miR-208a, consistent with the lack of seed match sequence for this or other PDEs (Figure S6A–E).

To experimentally validate if 3′UTR of PDE4D is a miR-208a target, the 3′UTR sequence of PDE4D was placed in frame and downstream of the GFP cDNA (Figure S7A). Transfection of miR-208a + GFP-PDE4D3′UTR into HEK cells resulted in the significant reduction of GFP protein as determined by Western blotting (Figure S7B, lane 5). Transfection of miR-208a + GFP-PDE4D3′UTR + scrambled antagomir into HEK cells also resulted in the reduction of GFP (Figure S7B, lane 7). GFP expression was not altered when miR-208a + antagomir-208a + GFP-PDE4D3′UTR were co-transfected into HEK cells, and is evidence of the inactivation of miR208a by antagomir-208a (Figure S7B, lane8). Moreover, GFP expression did not change in any of the controls used (Figure S7B, boxed). Western blots were quantified and summarized as shown in Figure S7C and are evidence that miR-208a is specifically engaged with the 3′UTR of PDE4D to suppress GFP.

### Effects of miR-208a on PRKAR1α, THRAP1 and miRs harboring PDE4D recognition sequence

Despite the positive effects of miR-208a on cardiac function, it is unclear, however, whether this cardiac specific miR has effects on other target genes or miRs. Thus, we sought to investigate further the mechanism by which miR-208a mediates enhanced cardiac myocyte performance. We reasoned that miR-208a may amplify the cAMP/PKA pathway by regulating additional target genes. To test this, we focused on potential genes that activate the PKA pathway. Interestingly, miR-208a negatively regulated the expression of cAMP dependent regulatory, type I, alpha (PRKAR1α), which is major regulatory subunit of PKA (Figure S8). The down regulation of PRKAR1α drives releases of the PKA catalytic subunit. Thus, miR-208a relieves this inhibition causing release of catalytic subunit (PRKCA) leading to PKA activation.

The target scan prediction algorithm shows that PRKAR1α 3′UTR has two seed match sites for the miR-208a seed sequence and is conserved between human and dog, albeit less conserved in rodents (Figure S8A). The non-conserved sequences are indicated in black letters. Despite this, miR-208a overexpression in adult cardiac myocytes decreased levels of the PRKAR1α protein compared to miR-208a mutant and untreated controls (Figure S8B,C). Similarly the level of PRKAR1α was reduced in miR-208a transgenic mice compared to control and 3 weeks dox treated transgenic mice (Figure S8D,E). We determined that treatment of miR-208a transgenic mice with dox for 3 weeks brings the level of miR-208a to a basal level. Owing to less conservation of the recognition sequence of PRKAR1a in rodents, we hypothesized that the regulation of PRKAR1a by miR-208a is indirect. Moreover, because miR and target mRNA interactions are complex, it could be that suppression of PRKAR1α is mediated by a non-canonical miR/mRNA interaction. Thus the possibility of non-canonical interactions could not be excluded. For instance, it has been shown that although most published interactions involve the canonical miR seed and mRNA seed match, from an emerging paradigm the possibility of many other modes of miR bindings beyond the seed have been detected, including 3′ compensatory, 3′ supplementary, imperfect centered, perfect centered, G-bulge and others^35^,^36^,^37^. From bioinformatics, we find that the less conserved second site appears to have a 3′ region supplementary for human and dog or 3′ compensatory for rodents (Figure S8A, light blue). Taken together suppression of PRKAR1a could be mediated by 3′ compensatory for rodents or by some indirect effects. To serve as a positive control, the 3′UTR sequence of THRAP1 was placed in frame and downstream of the GFP cDNA (Figure S9). Transduction of ventricular myocytes with adenovirus harboring GFP-THRAP13′UTR resulted in the expression of a strong GFP signal. Transduction of ventricular myocytes with AdmiR-208a + AdGFP-THRAP13′UTR resulted in significant reduction of GFP protein as determined by live fluorescence imaging. Interestingly dilution of admiR-208a to 1: 10^5^ did not reduce GFP expression suggesting dose-dependent effect.

To investigate the possibility of miR-208a effects on other miRs, we focused on several key miRs that are down-regulated in heart failure and thus important for cardiac function^38^,^39^,^40^,^41^,^42^,^43^. Interestingly, the expression levels for miR-30b, miR-26b, miR18a and miR-101 were increased in miR-208a transgenic hearts (Figure S10A–D). Further, in silico prediction algorithm analysis showed that these miRs also have a seed sequence that targets the PDE4D 3′UTR. These observations suggest that miR-208a enhances cardiac function in concert with additional key cardiac expressed miRs. Similar experiments in adult rat ventricular cardiac myocytes treated with adenovirus expressing miR-208a showed a similar effect on miR-101, but not on other miRs suggesting species variation (Figure S10E–H).

### MiR-208a increases phosphorylation of cTnI and PLN

**Figure 3:**
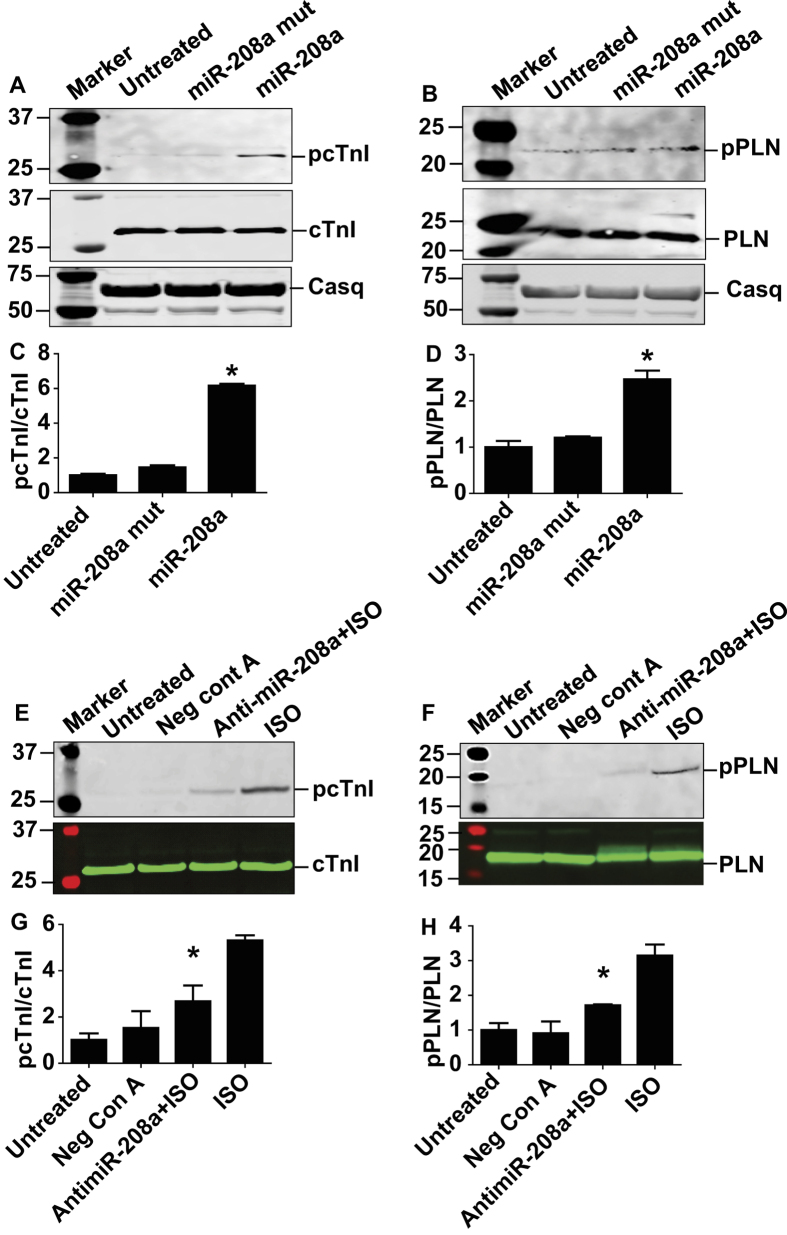
MiR-208a increases phosphorylation of cTnI and PLN in adult cardiac myocytes. Western blot analysis of key phosphoproteins regulating SR calcium handling, relaxation and contractility after miR-208a transduction. Here, an increase in phosphorylation of cTnI (PKA sites Ser23/24) (**A,C**) and increase in phosphorylation of PLN (PKA site Ser16) (**B,D**) as compared to untreated control and miR-208a mutant was observed. MiR-208a did not affect total content of cTnI and PLN (**A,B**). Calsequestrin was not altered with either miR-208a or miR-208a mutant transduction or non-transduced control myocytes (**A,B**) and is used as loading control and for quantification of pcTnI and pPLN in each sample. Acute suppression of miR208a expression by anti-miR-208a reduced ISO-induced phosphorylation of cTnI and PLN (**E–H**). Data are shown as mean +/− SEM, n = 3–5 *P < 0.05.

Next we sought to determine the mechanism by which miR-208a-mediated suppression of PDE4D and PRKAR1α causes enhanced cardiac myocyte performance. Because miR-208a inhibits PDE4D and PRKAR1α to amplify the cAMP/PKA pathway, we examined the phosphorylation status of known key PKA-site specific cardiac phosphoproteins that regulate myofilament and sarcoplasmic reticulum Ca^2+^ handling function (Fig. 3A–D). Notably, increased miR-208a expression led to an increase in phosphorylation of cardiac troponin I (cTnI) at PKA sites Ser23/24 and increased the PKA-specific phosphorylation site of phospholamban (PLN) (Ser16), as compared to untreated control and miR-208a mutant (Fig. 3C,D). Total cTnI and PLN content did not change by miR-208a treatment and were used as loading control and for the normalization of each sample for quantification of relative expression of phosphorylated cTnI and PLN (Fig. 3A–D). Expression of calsequestrin was not altered by miR-208a. As further validation of a cause/effect relationship, we acutely suppressed miR-208a expression using a miR-208a power inhibitor: a locked nucleic acid (LNA) enhanced antisense oligonucleotide directed against miR-208a. Then, to broaden the cardiac phosphoprotein dynamic range, we used 3 nM isoproterenol to increase phosphorylation of cTnI and PLN and evaluated the reduction in ISO-induced phosphorylation of cTnI and PLN following miR-208a inhibition. Here, miR-208a inhibition caused significant hypo-phosphorylation of the Iso-induced cTnI and PLN (Fig. 3E–H).

### Titration of miR-208a levels and effects on PDE4D and phosphoproteins *in vivo*

**Figure 4:**
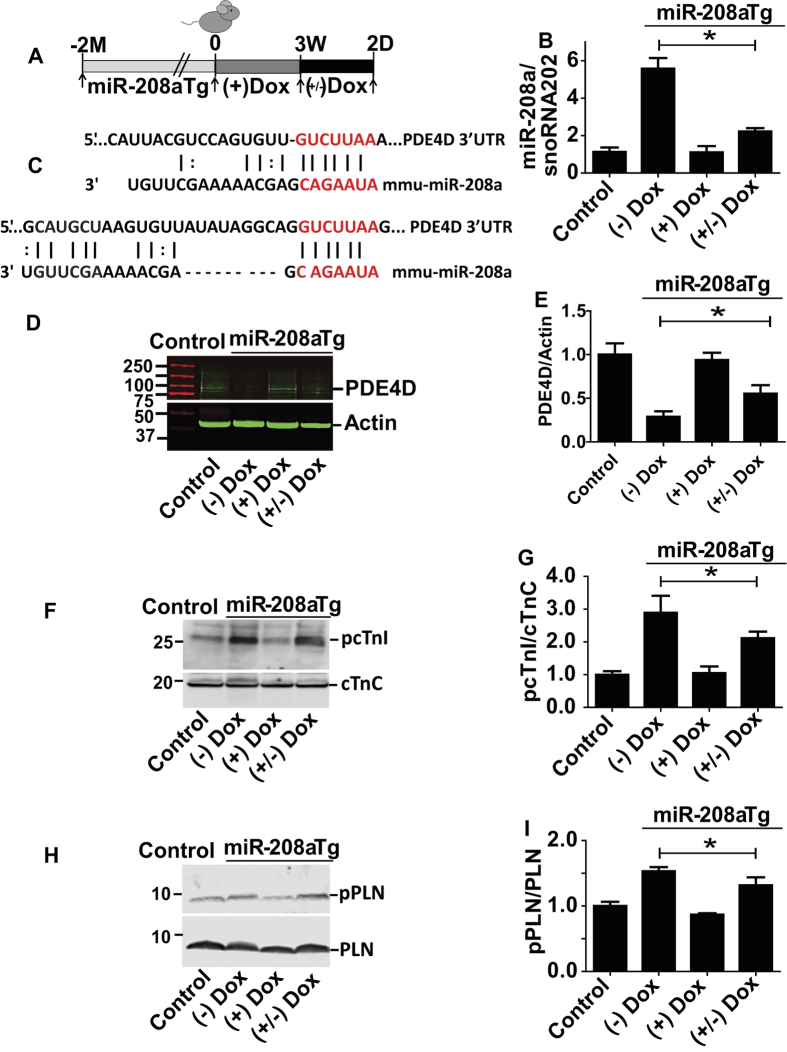
*In vivo* titration of miR-208a increases cardiac phospho-substrates by suppressing PDE4D. Experimental timeline for transgenic mice (**A**) and summary of titration of miR-208a levels *in vivo* (**B**). Two sites showing alignment highlighting the seed match sequences of mouse PDE4D 3′UTR (red) that is complementary for the seed sequences of miR-208a (**C**). The PDE4D content (**D,E**), the phosphoprotein content for cTnI (**F,G**) and PLN (**H,I**) are shown with representative Westerns and summary plots. Data are shown as the means +/− SEM. N = 3 *P < 0.05. Abbreviations, (+) Dox = dox treated, (−) Dox = no dox, (+/−) Dox = dox treated for 3 weeks and dox removed for 2 days.

For titration of miR-208a levels *in vivo*, we used a dox inducible miR-208a expression system under the control of αMHC promoter where miR-208a is specifically expressed in the heart^25^. MiR-208a double transgenic mice (with overall average expression of 5-fold increased miR-208a) were given doxycycline (Dox) in the drinking water for three weeks, which pilot studies had determined was sufficient time to decrease the transgenically overexpressed miR-208a to baseline levels. Dox was then withdrawn for two days to acutely re-activate the miR-208a transgene (reactivation ranging from 1.5 to 2.5-fold over baseline) (Fig. 4B) for assessment of PDE4D content (Fig. 4D,E), and phosphoprotein levels (Fig. 4F–I). Compared to controls, myocardial PDE4D and PRKAR1α levels were reduced in transgenic mice (Figs 4D,E and S8D,E), restored by Dox-mediated Tg suppression, and then acutely depressed upon re-activation of the miR-208a transgene *in vivo* (Fig. 4D,E). Consistent with these findings, the PKA-site dependent phosphorylation status of cTnI and PLN was high in transgenics, reduced to baseline levels after three weeks of Dox treatment, and then acutely increased after removing Dox treatment for two days (Fig. 4F–I).

### MiR-208a enhances heart performance *in vivo*

**Figure 5:**
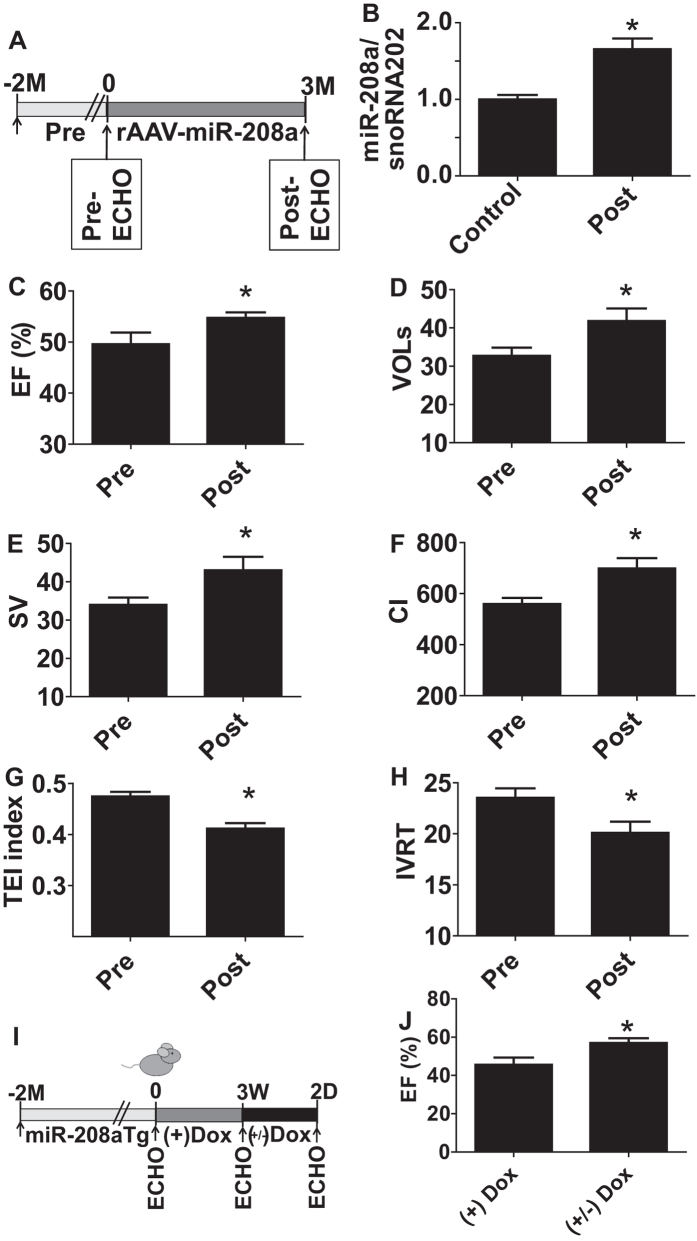
MiR-208a enhances contractility and relaxation function *in vivo*. Experimental timeline for rAAV-miR-208a delivery (**A**) and summaries of miR-208a expression (**B**) and echocardiography-based EF (**C**), VOLs (**D**), stroke volume (**E**), Cardiac index (**F**), TEI index (**G**) and IVRT (**H**). Data are shown as mean +/− SEM, n = 13–14 mice; *P < 0.05 compared with the pretreatment group. Experimental timeline for *in vivo* titration of miR-208a in transgenic mice (**I**) and summary of echocardiography-based cardiac function following miR-208a titration (**J**). Data are shown as the mean +/− SEM, n = 27 (**J**); *P < 0.05.

We hypothesized that acute *in vivo* expression of miR-208a would have beneficial effects on heart performance. As a first approach, we used rAAVmiR-208a *in vivo* cardiac delivery and this increased myocardial miR-208a expression 1.5-fold over baseline (Fig. 5B). Echocardiography showed a significant increase in heart performance (Fig. 5C–H). However, owing to transgene variable slow kinetics of rAAV transgene expression (~10 weeks), it can be challenging to use this system for dissecting acute (days) from chronic effects (months) of increased miR-208a expression. To conduct with greater temporal precision the titration of miR-208a levels *in vivo*, we used a dox inducible miR-208a expression system under the control of αMHC promoter where miR-208a is specifically expressed in the heart^25^. Echocardiography showed an improved LV ejection fraction function upon Dox-mediated titration of miR-208a content in Tg animals *in vivo* (Fig. 5I,J).

## Discussion

We report evidence of a new signaling mechanism of positive inotropy whereby a cardiac myosin-embedded miR targets suppression of cAMP-specific 3′,5′-cyclic phosphodiesterase 4D and protein kinase A regulatory subunit I alpha (PRKAR1α). Findings show that with acute increases in miR-208a, PDE4D and PRKAR1α protein content is significantly reduced in cardiac myocytes causing increased PKA site-dependent phosphorylation of troponin I (cTnI) and phospholamban (PLN). Several miRs harboring PDE4D recognition sequence are also increased contributing to reduced PDE4D protein content. The physiological effects of PLN/cTnI phosphorylation are sufficient to account for enhanced myocyte performance by miR-208a expression. The fast turnover rate of PDE4D (4 hours)^34^ is an essential element enabling the timely responsivity of this miR-mediated positive inotropy pathway. Previous reports on cardiac miRs have mainly focused on the long-term myocardial growth/remodeling outcomes associated with this novel class of RNAs^25^,^26^. For example, miR-208 deficient animals showed no fibrosis or hypertrophy of cardiac myocytes in response to pressure overload induced by TAB. Although βMHC expression is reduced, other hypertrophy markers such as ANF and BNP are still increased in this chronic model, suggesting complex biological processes mediated by miR-208 in the heart^26^. Furthermore, therapeutic inhibition of miR-208a improves cardiac function and survival during heart failure. However, inhibition of βMHC expression required long-term treatment by antisense directed against miR-208a^27^. This is consistent with our observation that acute titration of miR-208a does not alter myosin gene isoforms.

Our findings that miR-208a-directed regulation of PDE4D content, signaling and contractile function are independent of heart growth/remodeling or altered host myosin gene expression effects are consistent with the comparatively slow turnover rates of host gene cardiac myosin effects (~ 5.4 days)^44^. Collectively, these findings support a potential new mechanistic signaling pathway for short-term enhancement of myocardial performance. In terms of pathophysiological significance, increased heart performance in the short-term could be beneficial in acute disease presentations such as in cardiogenic shock.

**Figure 6:**
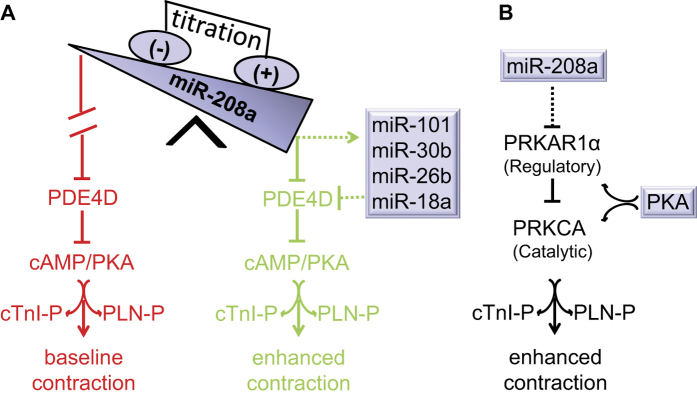
Working model: miR-208a titration and contractile function. Cardiac expression of miR208a suppresses PDE4D through engagement of the seed sequence of miR-208a to the seed-match sequence of PDE4D 3′ UTR. MiR-208a also up-regulates other miRs targeting PDE4D. Suppression of PDE4D by miR-208a and other miRs promotes increased phosphorylation of cTnI and PLN thereby enhancing cardiac function (**A**, Green). Baseline contractility is depicted in red (**A**). Suppression of regulatory subunit of PKA (PRKAR1α) by miR-208a leads to active catalytic subunit (PRKCA) and PKA activation (**B**). This concerted amplification of PKA signaling in turn increases phosphorylation of cTnI and PLN thereby enhancing cardiac function (**A**, green and **B**). The dotted line indicates indirect regulation.

A working model of miR-208a-mediated positive inotropy is presented in Fig. 6. Here acute titration of miR-208a expression above basal levels suppresses PDE4D (Fig. 6A) and PRKAR1α (Fig. 6B) to facilitate cAMP/PKA-dependent phosphoprotein signaling in cardiac myocytes. In concert, miR-208a up-regulates several cardiac expressed miRs that harbor the PDE4D site, including miR-101, miR-30b, miR-26b and miR18a to amplify suppression of PDE4D. This observation is consistent with the view that a single miR regulates several genes and vice versa. MiR-208a suppresses PDE4D through engagement of the 3′ UTR seed match sequence of PDE4D resulting in increased phosphorylation of cTnI and PLN, thereby enhancing cardiac function. Moreover, acute suppression of miR-208a increases PDE4D and reduces cTnI and PLN phosphorylation. The PLN-Ser 16 site detected here is unique to PKA and no other kinases^45^, consistent with miR-208a functioning through the cAMP/PKA pathway as mediated by PDE4D suppression. The effecter arm of this model is supported by a large body of literature demonstrating improved cardiac performance by PKA-mediated phosphorylation of cTnI and PLN^28^,^29^,^30^,^46^,^47^. Interestingly, a recent study^48^ has demonstrated that gain of miR208a function through transfection of miR-208a increased levels of cAMP and PKA, further supporting the involvement of miR-208a in cAMP/PKA signaling pathway for which our study now provides the molecular mechanism. In summary, through miR-208a gain/loss of function studies we posit a strong causative pathway has been established. It will be interesting in future studies to further test this working model (Fig. 6), including performing additional gain/loss of function studies by targeting PDEs, sarcomeric and SR target proteins *in vivo*.

Our findings may also help explain how sustained increases in miR-208a levels might be detrimental to heart function^25^. It is known that selective pharmacological inhibition of PDE4 or PDE4D gene ablation can enhance cardiac contractility, at least for a short period of time^49^. Chronically, however, PDE4D deficiency leads to deterioration in heart function^50^,^51^. The mechanism of positive inotropy observed by acute increased expression of miR-208a is consistent with PKA-site dependent cTnI phosphorylation that increases cross-bridge cycling rate and enhances unloaded shortening velocity^52^,^53^. It is also consistent with the key role of cTnI phosphorylation in positive inotropy resulting from β-adrenergic stimulation^54^. Increased phosphorylation of cTnI and PLN directly facilitates fast thin filament inactivation and Ca^2+^ sequestration, respectively, to improve relaxation performance^55^. That cTnI/PLN phosphorylation is sufficient to account for enhanced myocyte function by miR-208a does not preclude other pathways mediated by miR-208a, or other miRs that could also affect heart performance. Our genetic strategy using miR-208a to inactivate PDE4D provides insight into the function of PDE4D proteins as compared to non-selective pharmacological inhibition of PDE4D.

Recent evidence of PDE4D microdomains in cardiac myocytes that influence PLN, but not RYR or L-type Ca^2+^ channels^49^, is supported by our finding that the Ca^2+^ transient amplitude is unaffected by miR-208a. Our model (Fig. 6) is consistent with the reduced contractility and increased left ventricular dimensions at baseline and during stress in miR-208a deficient mice^26^. It is also consistent with recent work in DCM patients where miR-208a levels are reduced and the miR-208a target PRKAR1α is up-regulated^24^. Furthermore, cardiac specific deletion of PRKAR1α is embryonic lethal with a DCM phenotype and with up-regulation of PKA^56^. Owing to reduced conservation of the recognition sequence of PRKAR1a in rodents, we hypothesize that the regulation of PRKAR1a by miR-208a is indirect. Moreover, because miRs and target mRNA interactions are complex, it could be that suppression of PRKAR1α is mediated by a non-canonical miR/mRNA interaction. For instance, it has been shown that although most published interactions involve the canonical miR seed and mRNA seed match, from emerging paradigms the possibility of other modes of miR binding beyond the seed have been defined, including 3′ compensatory, 3′ supplementary, imperfect centered, perfect centered, G-bulge, and others^35^,^36^,^37^. For example, pairing to the 3′ region of the miR can supplement a 7–8mer match when 3–4 consecutive additional Watson Crick base pairing occurs at positions 13–16 or 13–17 of miR, termed 3′ supplementary^57^,^58^,^59^. Whereas it can also compensate for a single-nucleotide bulge or mismatch in the seed region when additional Watson Crick base pairing occurs at positions 13–16 or 13–17 of the miR, compensating for the mismatch in the seed region, and termed 3′ compensatory^57^,^58^,^59^. As illustrated in Figure S8A light blue, PRKAR1a suppression could be mediated by these mechanisms. Taken together, a combination of non-canonical miR/mRNA interactions, and/or indirect effects, mediates the PRKAR1α suppression in rodent myocytes. Moreover, targeted miR-208a suppression and consequent reduced phospho-cTnI/PLN content may account for the earlier observation that loss of cardiac specific miR-208a and cardiac enriched miRs leads to dilated cardiomyopathy and heart failure^16^,^17^,^23^,^24^,^60^,^61^.

Collectively, our findings support the hypothesis that both the duration and extent of altered cardiac miR-208a expression and its targets are critical parameters in modulating myocardial performance. Based on our findings, chronic miR-208a up-regulation would suppress PDE4D in the long-term, leading to potentially inappropriate heightened myocyte performance by altered PKA activation. This, in turn, could account for arrhythmias reported in transgenic mice that chronically over-express miR-208a^25^. This is supported by previous PDE4D ablation studies shown to be proarrhythmic^50^ and heart specific ablation of PRKAR1α is shown to cause DCM via inappropriate PKA activation^56^. In addition, chronic inotropic therapy in end-stage heart failure is well known to be associated with negative outcomes, including increased mortality^62^,^63^. Taken together, we propose acute increases in miR-208a would be beneficial to cardiac performance but would have untoward effects in the setting of chronically increased expression. While fine details in dose and timing remain, our results suggest that both the duration and level of miR-208a expression are key parameters in defining myocardial physiological outcomes^24^,^25^,^26^,^56^,^60^,^61^,^64^,^65^.

Phophodiesterases (PDEs) are essential in cell signaling by mediating the degradation of cyclic nucleotides. The mammalian genome contains more than 20 PDE genes that are subdivided into 11 PDE families on the basis of sequence homology, substrate specificity, and inhibitor sensitivity^66^. PDE inhibitors hold promise to improve cardiac performance, cell survival during ischemic heart attack, heart failure and during cardiopulmonary bypass surgery^67^. The PDE4 family is composed of four genes: PDE4A, PDE4B, PDE4C, and PDE4D and they are all expressed in the heart (except for PDE4C)^31^. These encode more than 20 isoforms through the use of different promoters/alternative splicing^68^,^69^. Because of this isoform diversity, it is important to verify the specificity of miR-208a for PDE4D. Accordingly, the expression levels of several cAMP PDEs were profiled by qRT-PCR (Figure S3A–I). Consistent with the lack of seed match sequence the transcript level of PDE4A, PDE4B, PDE1A, PDE3A, PDE8A and PDE8B were not reduced by miR-208a. However, both PDE4D3 and PDE4D9 were reduced, consistent with the presence of seed match sequence for miR208a. Unexpectedly, PDE8A mRNA increased following miR-208a expression (Figure S3H). Others report that Ca^2+^ transients are increased in PDE8A null (PDE8A^−/−^) ventricular myocytes during β-adrenergic signaling activation^70^. In addition, Ca^2+^ spark activity is higher in PDE8A^−/−^ than in WT myocytes. We speculate that miR-208a may prevent heightened spark activity by increasing the level of PDE8A transcript. PDE4D regulates cAMP/PKA in cardiac myocytes and is therefore an important regulator of cardiac contractility^49^. Recently, PDE4 activity was reported to co-precipitate with phospholamban, evidence that there is SR-associated PDE4 in human heart^71^. Reductions in cAMP/PKA signaling contribute to impaired cardiac function in heart disease patients^72^, making this an important target pathway for new therapeutic agents. Targeting inhibition of specific PDEs has great potential clinical value and further underscores the potential significance of miR-208a as a new PDE4D inhibitor.

To investigate the possible involvement of other miRs in miR-208a transgenic mice, we evaluated several key miRs that are down-regulated in heart failure and thus important for cardiac function. Interestingly, the expression levels of miR-101, miR-30b, miR-26b, and miR-18a were increased in miR-208a Tg mice. In previous works, miR-26b was shown to be anti-hypertrophic and prevented atrial fibrillation^38^,^39^, miR-30b had anti-fibrotic and anti-apoptotic effects during IR injury^42^,^43^, miR-18a prevented ECM remodeling^40^, and miR-101 prevented post infarct cardiac fibrosis and improved left ventricular compliance^41^. Taken together, these miRs could contribute to the results shown here. Because miRs are products of transcription, possibilities may exist for miRs to regulate other miRs. MiR-mediated miR regulation is the basis for amplification of small direct effects of miRs leading to defined phenotypes^73^,^74^. As examples, cross-regulation appears evident between members of the “myomiR” family^74^ and miR-378 regulates several cardiomiRs, including miR1a, miR92, miR98, miR145, miR-149, miR-212, miR-215, miR-224, miR501 and miR-504^73^. Taken together, our findings are consistent with these observations and add another layer of biological complexity to the miR mechanism of action.

In summary, we report evidence that cardiac α*MHC* gene-embedded miR-208a, in orchestration with other key cardiac expressed miRs, suppresses PDE4D and PRKAR1α to enhance cardiac myocyte function via PKA-site dependent phosphorylation of cTnI and PLN. Our findings of PDE4D/PRKAR1α-mediated positive inotropy and miR-25 as a negative inotrope^19^, help further establish miRs as unique targets for inotropic therapy. We envision the acute titration of miR-208a as a novel molecular signaling mechanistic pathway for direct modification of key performance enhancing phospho-substrates in the heart.

## Materials and Methods

### Cloning

To generate the pDC316miR-208a construct, mouse miR-208a precursor sequence with EcoRI and Bgl II sites was cloned into pDC316 shuttle plasmid cut with EcoR1 and Bgl II restriction enzymes. MiR-208a mutant was similarly generated as the wild type miR-208a, except that four nucleotides are mutated from the seed sequence of the wild type (Fig. 1). To generate the rAAV-miR-208a construct, the mouse miR-208a precursor sequence with EcoRI sites was cloned into pBH shuttle plasmid cut with the EcoR1 restriction enzyme. To generate the pDC316GFP-PDE4D construct, 3′ UTR of PDE4D containing the seed match sequence that is complementary to miR-208a and flanking sequence was cloned using SacI site at the 3′ of GFP cDNA in a pDC316GFP plasmid. PDC316GFP-PDE4D mutant was similarly generated, except four nucleotides were mutated from the seed match sequence of the wild type PDE4D. The PDC316GFP-THRAP1 was generated similarly. MiR-208a expression was confirmed by TaqMan microRNA quantitative RT-PCR (Applied Biosystems) analysis of transduced myocytes or Lipofectamin2000 (Invitrogen) transfected 293HEK cells. A custom TaqMan small RNA assay was used to establish the expression of the miR-208a mutant.

### Generation of adenoviral vectors

To generate recombinant adenovirus vectors harboring the miR-208a or miR-208a mutant, GFP-THRAP13′UTR, the shuttle plasmids pDC316-miR-208a or miR-208a mutant or GFP-THRAP13′UTR and Ad genomic plasmids were delivered by cotransfection to HEK 293 cells using the AdMax™ system (Microbix Biosystems). After homologous recombination, recombinant adenovirus, AdCMV-miR-208A or AdCMV-miR-208a mutant or AdCMV- GFP-THRAP13′UTR capable of being packaged but replication defective were generated. To harvest high titer recombinant virus, infected HEK 293 cells were collected and lysed by three cycles of freezing and thawing. After removing cellular debris, the supernatant of the viral lysate was stored at −20 °C. Southern blot analysis was used to identify recombinant adenovirus^75^.

### Isolation, primary culture and gene transfer of adult rat ventricular myocytes

All experimental protocols, animal procedures (experiments) used in this study were approved by the University of Minnesota’s Institutional Animal Care and Use Committee (IACUC). In addition all the experiments and animal handling were performed in accordance with guidelines and regulations approved by the University of Minnesota’s Institutional Animal Care and Use Committee (IACUC).

Female Sprague–Dawley rats (200 g) were anesthetized by inhalation of isoflurane. Hearts were mounted on a modified Langendorff apparatus, perfused for 5 min with Krebs–Henseleit buffer (pH 7.40 containing 1 mM CaCl_2_ (KHB with calcium), followed by KHB lacking added Ca^2+^ (calcium free KHB) for 5 min, and then with 60 ml of re-circulating KHB calcium free perfusate containing collagenase type II (0.3 mg/ml, Worthington) and hyaluronidase (0.15 mg/ml; Sigma) for 15 min. After increasing the Ca^2+^ in the digestion solution to 1 mM, and continuing the perfusion for 15 min, isolated ventricles were minced into pieces and gently shaken in digestion solution with occasional trituration using silanized pasteur pipets. Undigested ventricular tissue was removed using a 230-μm mesh sieve. The cell suspension was centrifuged, and the Ca^2+^ was increased to 1.75 mM after re-suspending pelleted cells in KHB with Ca^2+^ containing 2% BSA. After centrifuging cells again, they were resuspended in M199 culture medium containing 50 units/ml penicillin plus 50 μg/ml streptomycin (P/S), and 5% fetal bovine serum. Rod-shaped ventricular myocytes were plated onto laminin- coated coverslips in M199 + P/S + 5%FBS for 2 hr and then transduced with recombinant adenovirus containing either miR-208a or miR-208a mutant at 500 moi in 2 ml of serum-free media (M199 + P/S) with medium change every day.

### Pharmacologic inhibition of miR-208a via miR-208a power inhibitor

For acute suppression of miR-208a, adult ventricular cardiac myocytes were transfected with 50 nM of locked nucleic acid (LNA) enhanced antisense oligonucleotide directed against miR-208a (anti-miR-208a also called miR-208a power inhibitor) (Exiqon) using lipofectamin 2000 reagent (Invitrogen). As the basal level of pcTnI and pPLN is low in cultured ventricular myocytes, miR-208a power inhibitor transfected adult ventricular myocytes were treated with 3 nM isoproterenol (ISO) for 10–15 min to increase phosphorylation and to then evaluate reduction in ISO-induced phosphorylation of cTnI and PLN following inhibition of miR-208a. Finally, PDE4D content and phosphorylation of cTnI and PLN levels were determined in control and experimental myocytes by Western blotting. Pharmacologic suppression of miR-208a was assessed using TaqMan microRNA expression assay system (Applied Biosystems).

### Western blotting and indirect immunofluorescence

Control, miR-208a mutant and miR-208a virus-transduced ventricular myocytes were maintained in culture for two days and were collected from each cover slip in Ripa buffer and protein separated by SDS-PAGE. Protein samples from control and transfected 293 HEK cells were similarly prepared. Separated proteins were transferred to nitrocellulose membrane and blocked with 5% milk (w/v in TBST (Tris-Buffered Saline Tween-20) for 1 hr. Blocked samples were probed with primary antibodies specific for GFP raised in mouse (1:1000, Sigma), αMHC (BAG5), ßMHC (A4.951), MF20 each raised in mouse (1:5000, Developmental Studies Hybridoma Bank), p-cTnI (Ser23/24) raised in rabbit (1:1000, Cell Signaling), p-PLN (Ser16) raised in rabbit (1:1000, Upstate Biotechnology), cTnI (4C2) raised in mouse (1:2000, Advanced Immunochemical Inc.), PLN (A1) raised in mouse (1:1000, Upstate Biotechnology), Calsequestrin raised in rabbit (1:1000, Upstate Biotechnology). PDE4D (H-69), PDE5A (H-120) (Santa Cruz) and PDE4D (Protein Tech) and PRKAR1α (abcam) raised in rabbit were used at 1:500 dilution. Sarcomeric actin raised in rabbit (clone A2103, 1:5000; Sigma) was used to evaluate protein loading. The binding of primary antibodies was visualized by goat anti-rabbit (IRDye 800 conjugates) (Rockland Immunochemicals) or goat anti-mouse (Alexa fluor 680 conjugates) (Invitrogen) secondary antibodies (1:5000) and scanned with LI-COR Odyssey Infrared Imaging System (LI-COR Biosciences).

For indirect Immunohistochemistry, control and transduced myocytes were fixed in 4% PFA and permeabilized with 0.5% Triton X-100 in PBS. Permeabilized cells were blocked with 20% goat serum. Blocked myocytes were probed with primary antibodies specific for monoclonal anti-α sarcomeric actinin (clone EA-53) (1:1000, Sigma), PDE4D (SAB4502128) raised in rabbit (1:500, Sigma) and PDE5A (H-120) raised in rabbit (1:300, Santa Cruz). The binding of primary antibodies was visualized with goat anti-mouse IgG mAb conjugated to Alexa 488 (1:1000, Sigma), and Goat anti-rabbit conjugated to Alexa 594 secondary antibodies (1:1000). DAPI was used to visualize the nucleus. Representative myocytes were photographed using a Zeiss confocal microscope.

### Measurements of sarcomere length and intracellular calcium

Control, miR-208a mutant or miR-208a transduced myocytes were maintained in serum free M199 culture medium supplemented with P/S. Two days later, individual cover slips were transferred to a temperature-controlled chamber mounted on a Nikon microscope stage and the chamber was filled with M199 medium. A video-based detection system (Ionoptix, Milton, MA) was used to detect sarcomere length in intact myocytes. The cell chamber temperature was maintained at 37 °C, and myocytes were stimulated at 0.5 Hz. For intracellular Ca^2+^ measurements, myocytes plated onto laminin coated cover slips were loaded with the fluorescent Ca^2+^ indicator by incubating with fura 2-AM (2 μM, Invitrogen) in M199 medium for 10 min at room temperature, followed by incubation for 10 min in M199 medium alone. Fura 2 fluorescence was then measured in individual cells using Ionoptix after mounting the cover slips in a chamber (Warner Instrument) on the stage of the microscope and adding M199 with regular change until sufficient numbers of myocytes were recorded. The cell chamber temperature was maintained at 37 °C and myocytes were stimulated at 0.5 Hz.

### Titration of cardiac miR-208a *in vivo*

All experimental protocols, animal procedures (experiments) used in this study were approved by the University of Minnesota’s Institutional Animal Care and Use Committee (IACUC). In addition all the experiments and animal handling were performed in accordance with guidelines and regulations approved by the University of Minnesota’s Institutional Animal Care and Use Committee (IACUC).

Here, tet-off miR-208a Tg animals were generated by crossing C57/BL6 MHC-tTA-VP16 mice with tet-O-miR-208a mice^25^. The double transgenic mice overexpress miR-208a in the absence of Dox, while supplying Dox in mice drinking water shuts off the miR-208a transgene. To fully reverse the 4–5 fold increase in miR-208a levels in transgenic mice over baseline miR-208a levels, Dox was required in the drinking water for three weeks, and is in keeping with apparent turnover (12 days) of cardiac miR-208a *in vivo*^26^. Next, after the three weeks of Dox treatment, where the level of miR-208a reached the baseline levels, Dox was halted to acutely re-activate the miR-208a transgene *in vivo*. In these *in vivo* miR-208a titration studies, mice were subjected to functional and biochemical analyses, including levels of phosphoproteins and PDE4D. In parallel studies, rAAV was generated using a dual plasmid system and standard calcium phosphate transfection techniques in HEK293 cells. Virus-containing cells and supernatant were harvested, processed through microfluidization to lyse the remaining cells and purified over a heparin sulfate column. Next, rAAV-miR-208a was delivered by ultrasound-guided percutaneous injection directly to the left ventricle using a 30 3/4-gauge needle with 75 μl of injected volume. Animals were injected with 1 × 10^10^ plaque-forming units of rAAV-miR-208a. Cardiac function was determined by echocardiography. Gene expression was verified by TaqMan microRNA assay system after rAAV-mediated gene transfer.

## Additional Information

**How to cite this article**: Bedada, F. B. *et al*. Molecular inotropy mediated by cardiac miR-based PDE4D/PRKAR1α/phosphoprotein signaling. *Sci. Rep*. **6**, 36803; doi: 10.1038/srep36803 (2016).

**Publisher’s note:** Springer Nature remains neutral with regard to jurisdictional claims in published maps and institutional affiliations.

## Supplementary Material

Supplementary Information

Supplementary Figures
