# Supplementary material for: Molecular inotropy mediated by cardiac miR-based PDE4D/PRKAR1α/phosphoprotein signaling
Source: Sci Rep. 2016 Nov 11;6:36803. doi: 10.1038/srep36803 (PMC5105063; doi:10.1038/srep36803)
Supplement: Supplementary Information [file srep36803-s1.doc]

**Molecular inotropy mediated by cardiac miR-based**

**PDE4D/PRKAR1α/phosphoprotein signaling**

Fikru B. Bedada^1^, Joshua J. Martindale^1^, Erik Arden^1^ and Joseph M. Metzger^1^

^1^Department of Integrative Biology and Physiology, University of Minnesota Medical School, 6-125 Jackson Hall, 321 Church Street SE, Minneapolis, MN 55455 U.S.A

**Supplementary Materials**

**Material and methods**

**Morphometric measurements of myocytes**

For morphometric analysis, myocytes were transduced with miR-208a and miR-208a mutant virus using freshly isolated myocytes (day 0). Myocyte area was determined by image analysis using Image J1 software in control, miR-208a mutant and wild type miR-208a transduced myocytes. Mean cell area was determined for 100 cells in each of the treatment groups.

**Quantitative RT-PCR for miRs**

Total RNA was isolated from control and experimental myocytes using Trizol reagent (Invitrogen) following manufacturer’s instructions. The expression of miR-208a was assessed by TaqMan microRNA expression assay system that detects mature miR-208a using specific primers (Applied Biosystems). Similarly, the expression of miR-208a mutant was done using custom TaqMan small RNA assay system (Applied Biosystems). The assay system accurately quantifies mature miRs with the ability to discriminate between homologous miRs differing by a single nucleotide. Each miR-specific RT primer, miR-specific forward PCR primer, specific reverse PCR primer, miR-specific TaqMan MGB probe and TaqMan universal PCR master mix were purchased from Applied Biosystems. Quantification was done using two-step RT-PCR. In the reverse transcription (RT) step, cDNA was reverse transcribed from 100ng total RNA samples using specific miR primers and reagents from the TaqMan MicroRNA Reverse Transcription Kit. In the PCR step, PCR products were amplified from cDNA samples together with the TaqMan Universal PCR Master Mix. All qPCR assays were performed in duplicate on all control and experimental groups and normalized against control myocytes. Relative quantitation of gene expression was conducted according to the 2^-ΔΔCT^ method as described [^1^](#_ENREF_1). The SnoRNA 202 gene, which is closer in size (length) to miRs, was used as endogenous internal standard for expression analysis to determine the abundance of amplified target miRs within the same sample [^2^](#_ENREF_2). For cAMP PDEs quantification, we used the TaqMan Reverse Transcription Kit. PCR products were amplified from cDNA samples using the SYBR Green PCR Master Mix (Invitrogen). GAPDH was used as endogenous internal standard for PDEs expression analysis to determine the abundance of amplified target gene within the same sample as described [^1^](#_ENREF_1).

**Target gene validation**

For experimental validation, HEK cells were co-transfected with a construct containing either GFP alone, GFP+miR-208a, GFP+Scrambled antagomir, or GFP-PDE4D3’UTR as control. To test for suppression of GFP by miR-208a, HEK cells were co-transfected with GFP-PDE4D3’UTR+miR-208a or miR-208a+GFP-PDE4D3’UTR+scrambled antagomir. Additional controls for miR-208a-mediated GFP suppression in HEK cells were miR-208a+GFP-PDE4D3’UTR+antagomir-208a; GFP-PDE4D3’UTR+scrambled antagomiR, miR-208a mutant+GFP-PDE4D3’UTR and miR-208a+GFP-PDE4D3’UTR mutant. 48 hrs after co-transfection, samples were collected for Western blot based analysis of GFP expression. Relative expression of the GFP was obtained from Western blot analysis after normalization against GFP alone. Actin was used as loading control and for normalization of GFP protein in each sample.

**References**

1 Livak, K. J. & Schmittgen, T. D. Analysis of relative gene expression data using real-time quantitative PCR and the 2(-Delta Delta C(T)) Method. *Methods* **25**, 402-408, doi:10.1006/meth.2001.1262

S1046-2023(01)91262-9 [pii] (2001).

2 Mishra, P. K., Tyagi, N., Kundu, S. & Tyagi, S. C. MicroRNAs are involved in homocysteine-induced cardiac remodeling. *Cell Biochem Biophys* **55**, 153-162, doi:10.1007/s12013-009-9063-6 (2009).

**Supplementary figures:**

**
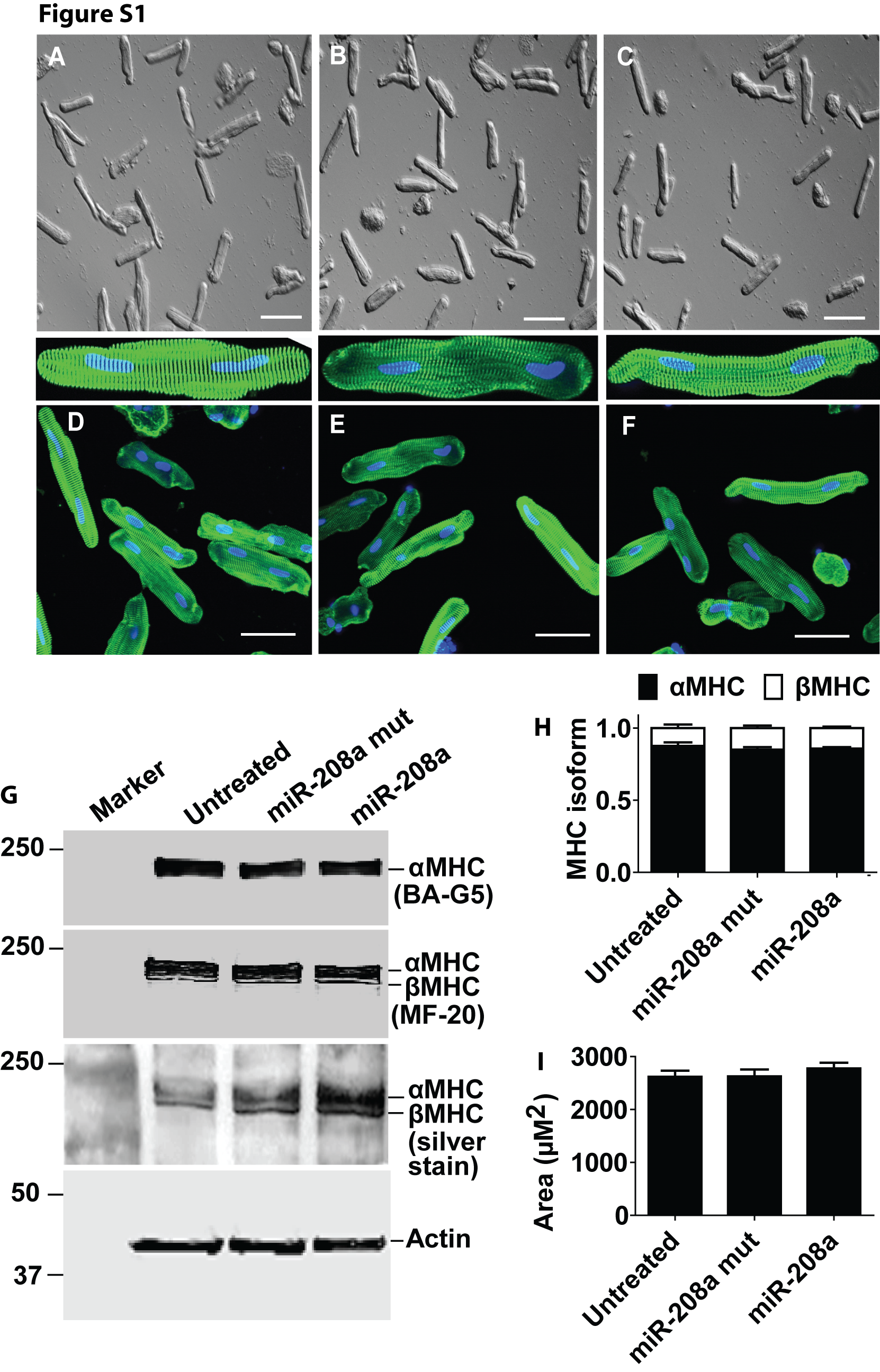

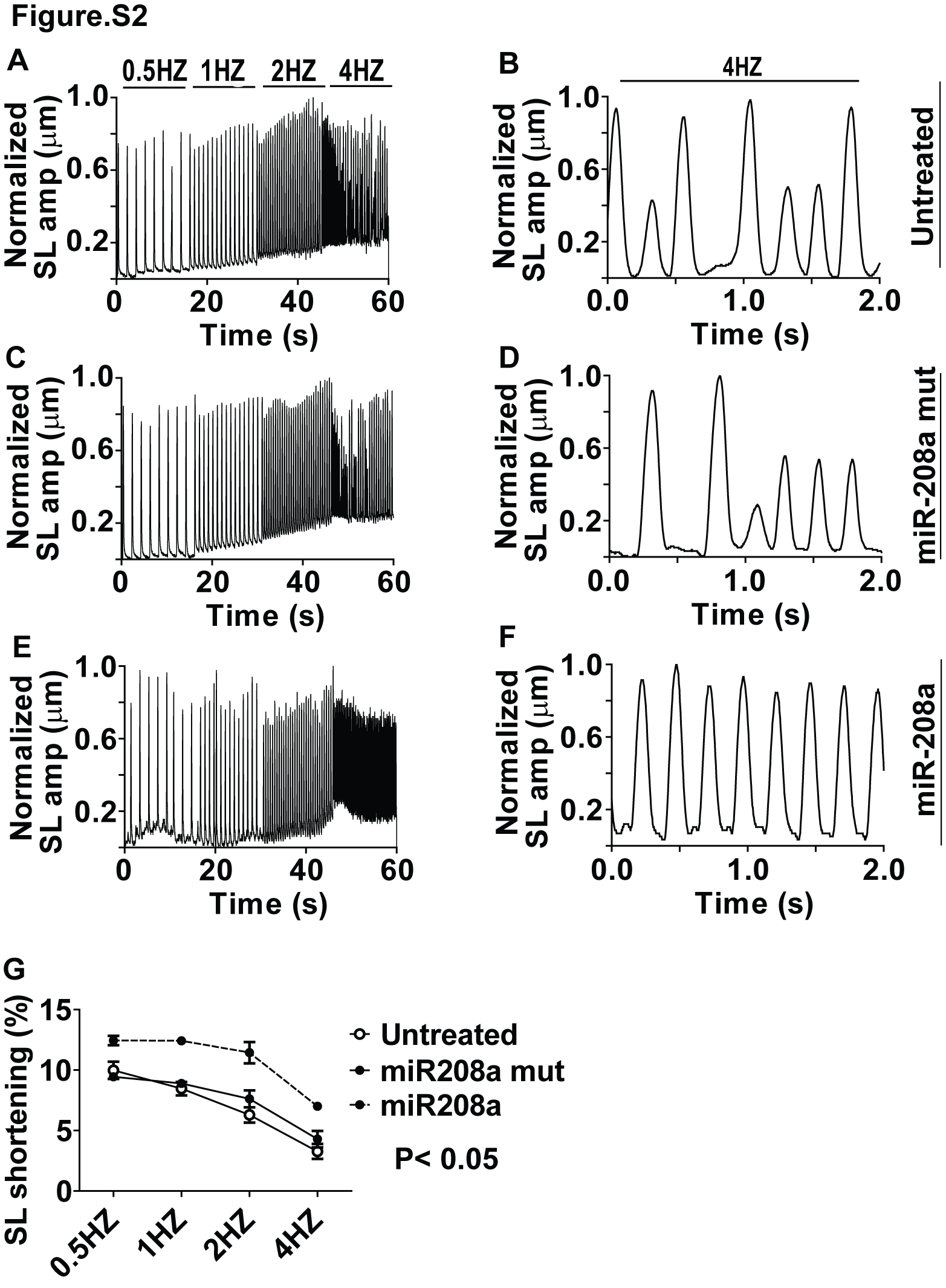

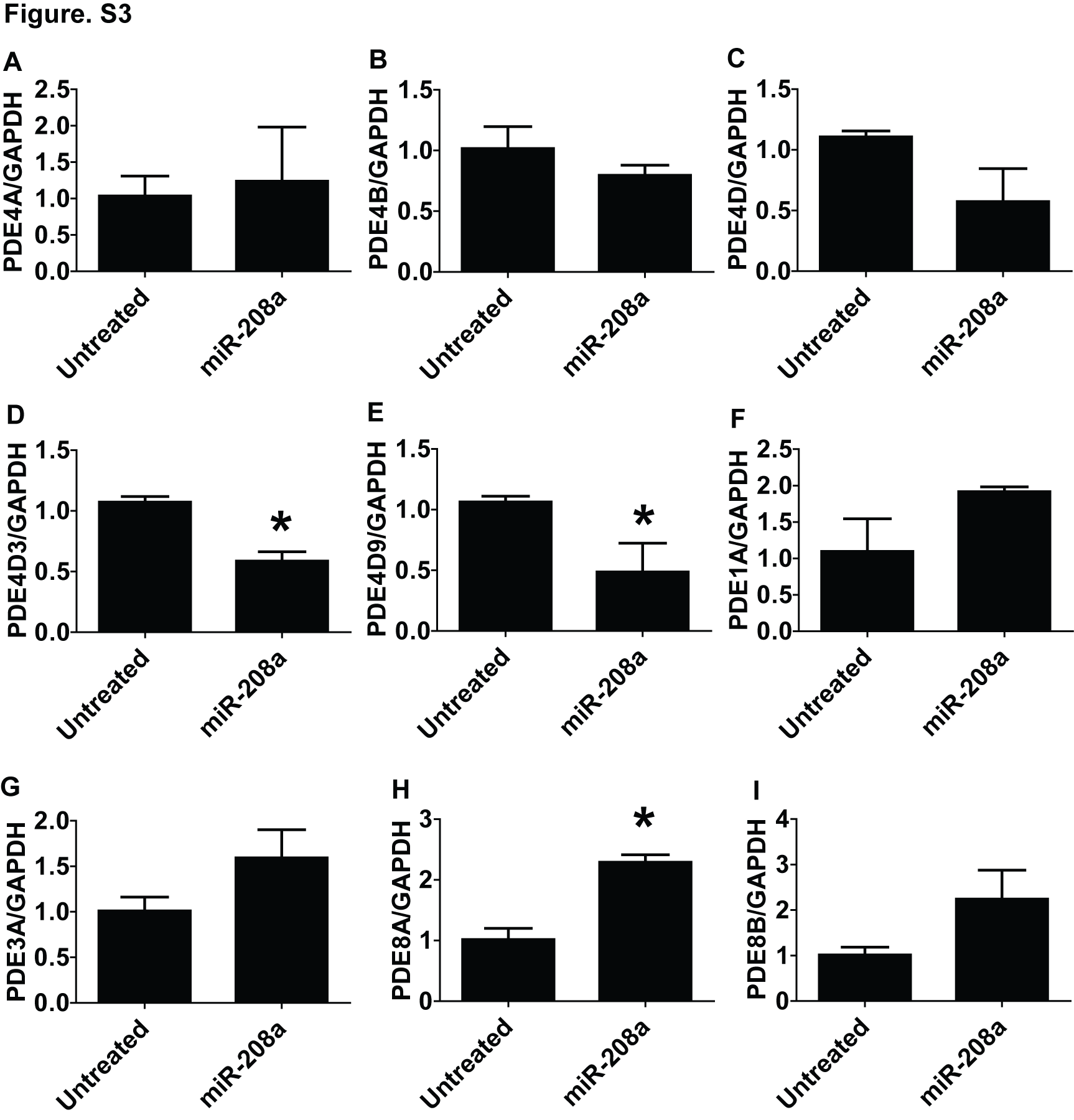

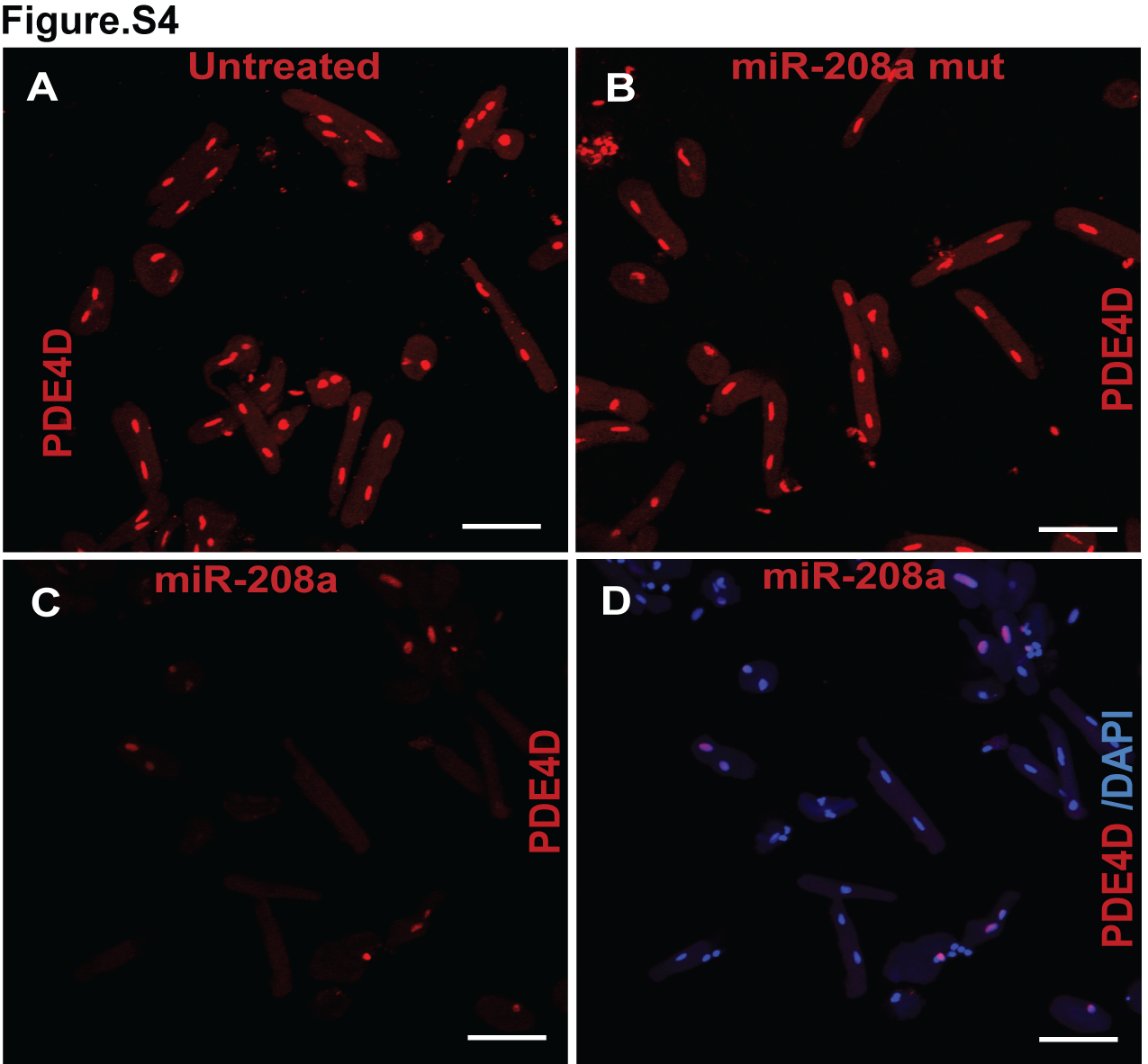

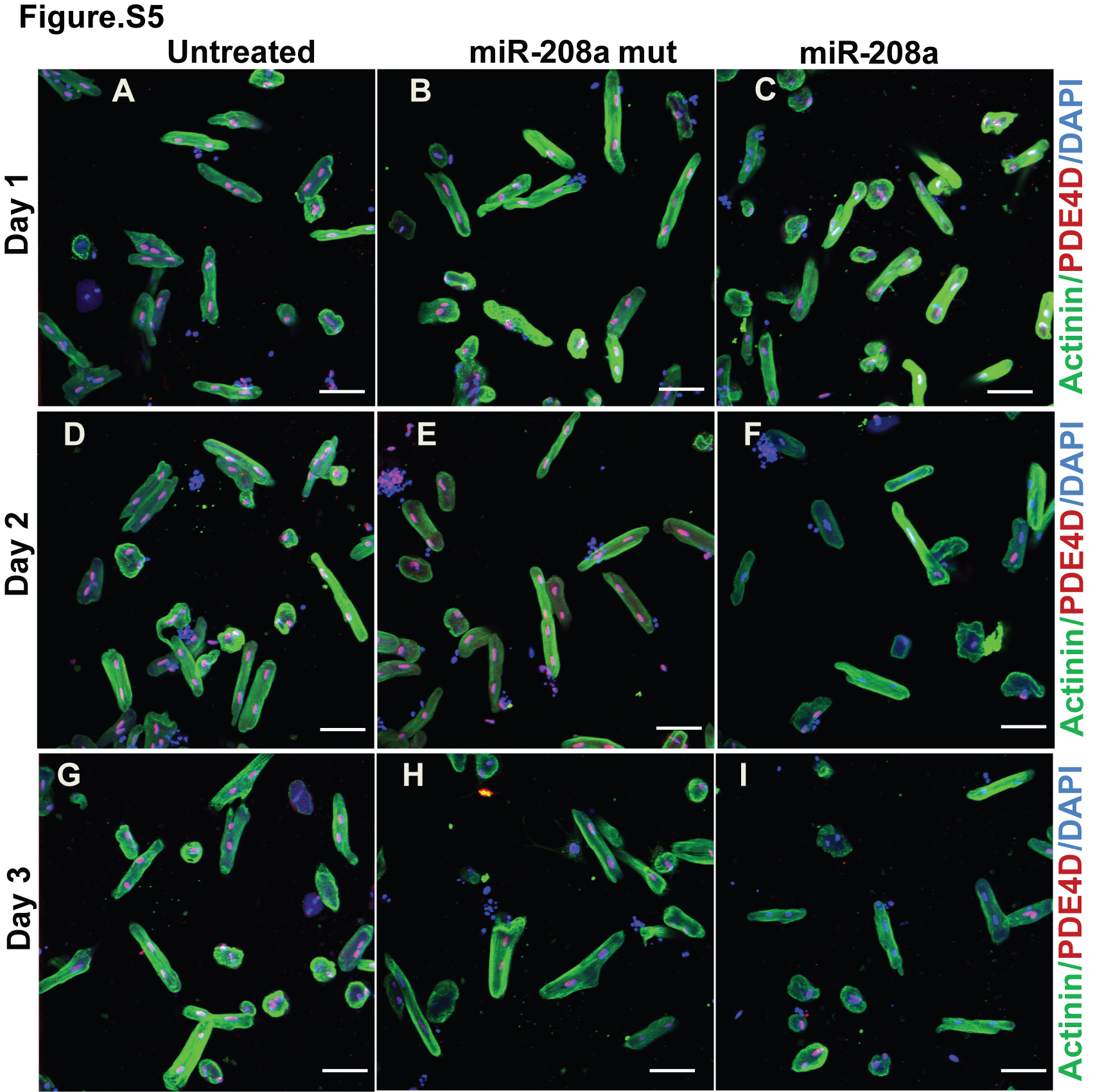

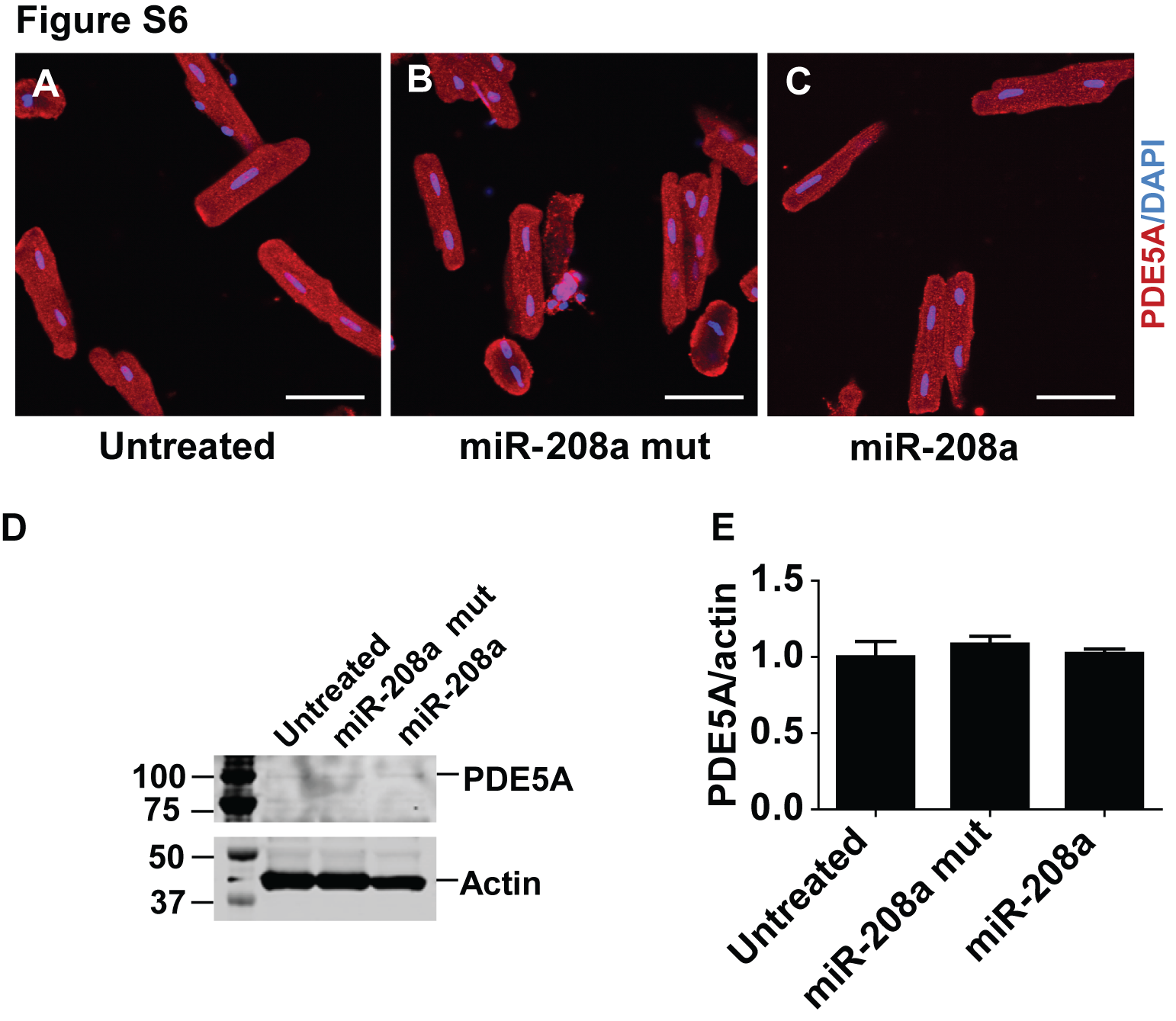

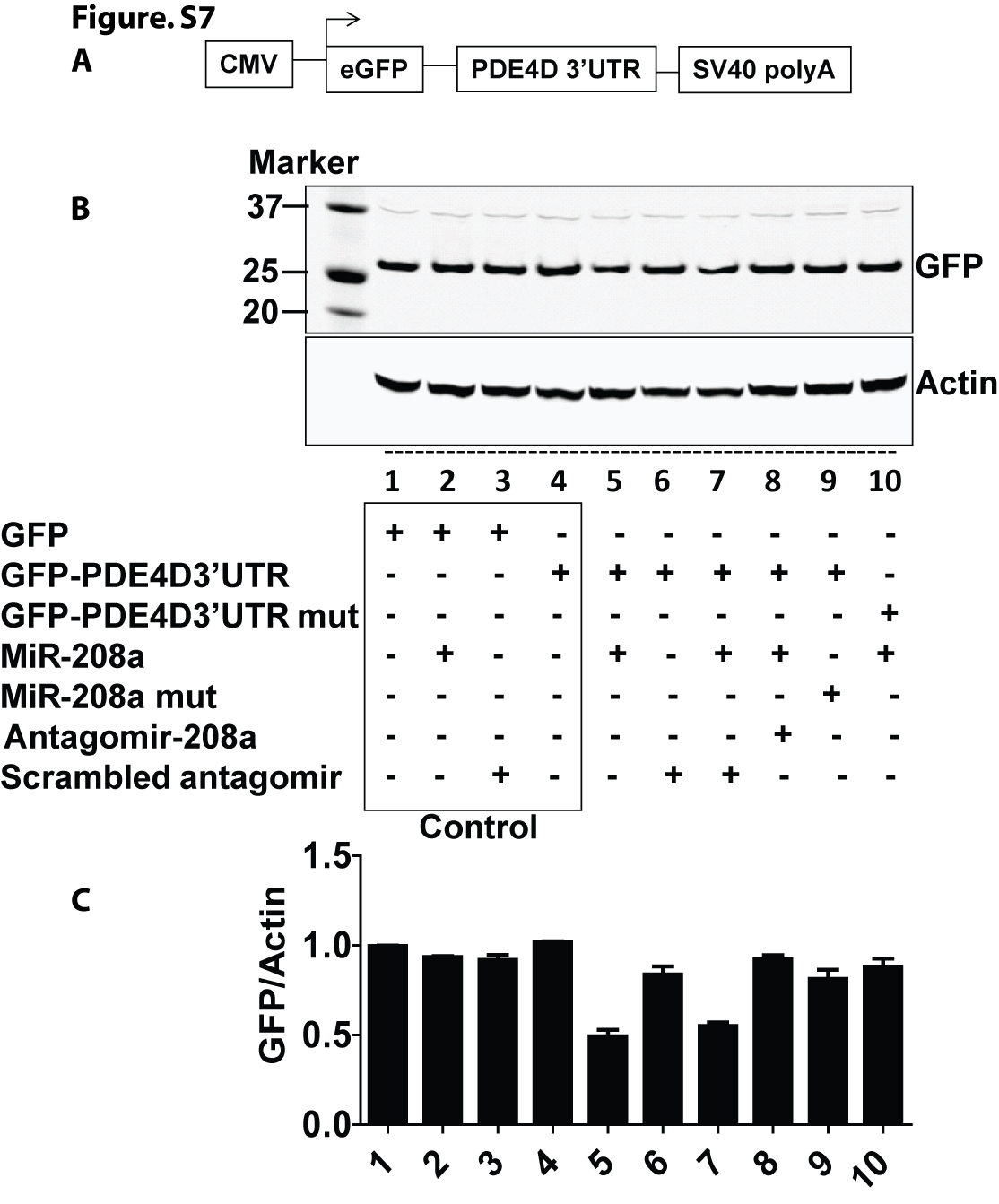

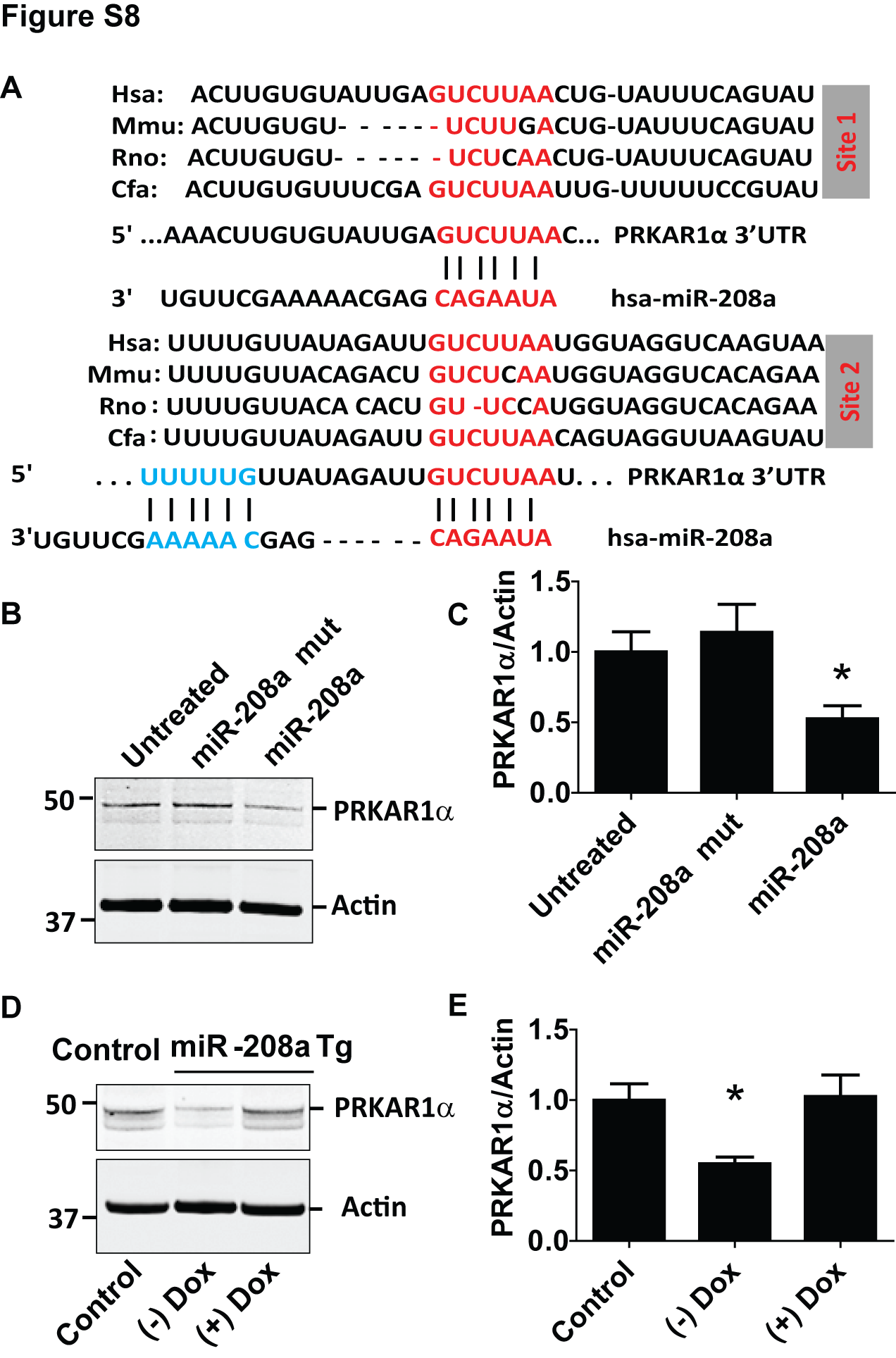

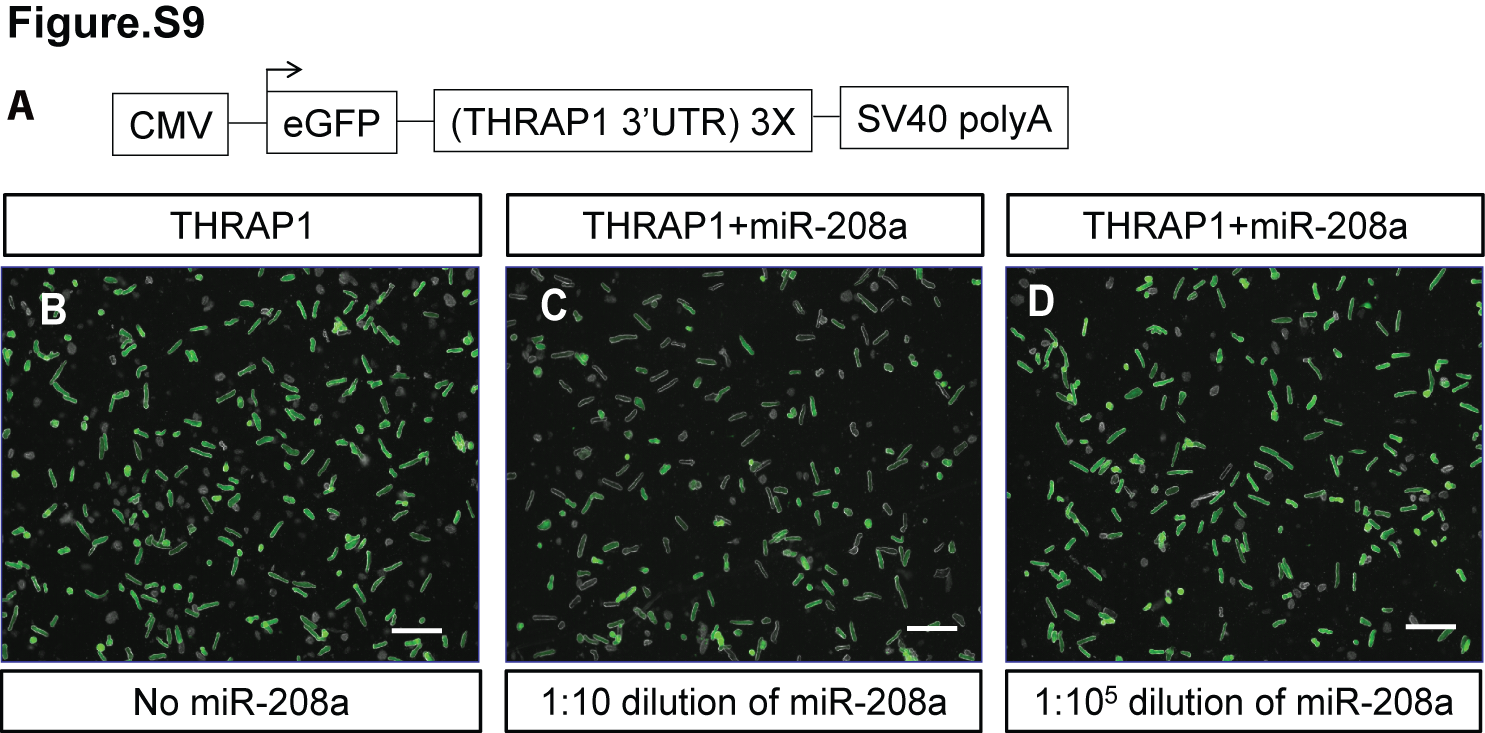

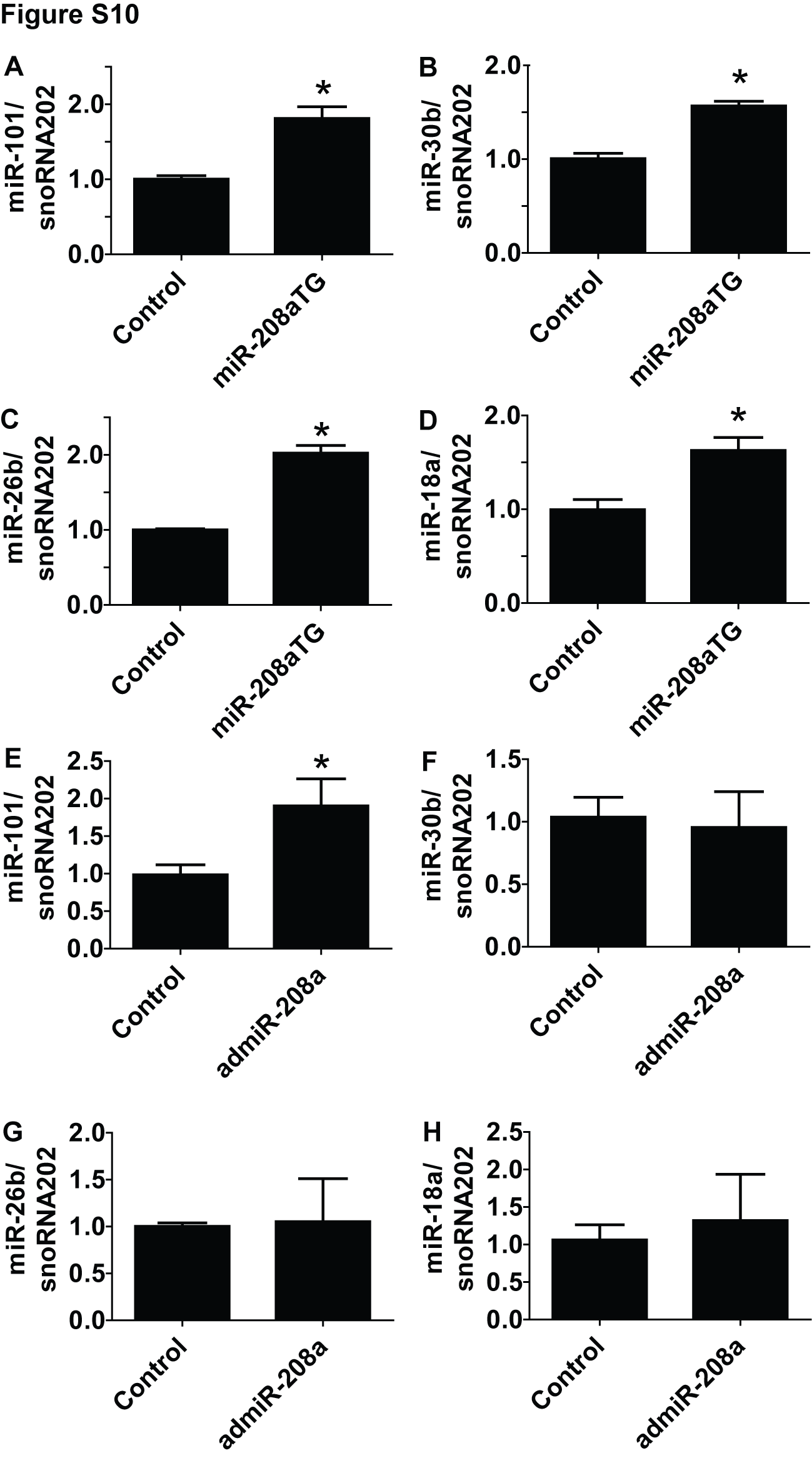
**

**Supplementary Legends**

**Figure S1. Acute gene transduction of miR-208a has no direct effect on adult myocyte morphology or myosin isoform expression pattern in vitro.**

Representative phase contrast images showing normal morphology of untreated (A), miR-208a mutant (B) and miR208a (C) transduced ventricular myocytes. Representative immunohistochemistry images of ventricular myocytes using sarcomeric actinin staining in untreated (D), miR-208a mutant (E) and miR208a (F) treated myocytes. Western blotting for αMHC, MF20 and silver staining (third panel) showing myosin isoform separation, actin showing loading control are depicted in (G). The original Western blots for these figures are shown in Figure S15. The cropped regions of the Western blots used in the manuscript are underlined in the original Western blot. The quantification of the relative expression of αMHC and βMHC is shown in panel H. Morphometric quantification of the size of the myocytes from panels (A,B,C) is depicted in panel (I). The phase contrast images for panels (A, B, & C) were taken with 20x objective confocal microscopy and bar shows scale of 100µm. The images for panels (D, E, & F) were taken with 40x objective Zeiss confocal microscopy and bar shows scale of 50µm.

**Figure S2**. **MiR-208a transduced adult myocytes have enhanced function during in vitro cardiac stress testing.**

MiR-208a, miR-208a mutant and untreated myocytes were subjected to stress testing by increasing the frequency of field stimulation (0.5, 1, 2 and 4 Hz) for untreated (A), miR-208a mutant (B) and miR-208a (E) myocytes. As shown in the right panels, stimulation at 4 Hz shows altered myocytes shortening SL traces for non-transduced control (B) and miR-208a mutant (D) whereas miR-208a transduced myocytes (F) are highly responsive during stress testing. Summary of SL during stress testing (G). Data are shown as mean +/- SEM, n = 5–10 myocytes. *P < 0.05 by One-way ANOVA.

**Figure S3. Effect of miR-208a expression on cAMP PDEs families.**

Quantitative RT-PCR based expression of PDE4 subtypes, PDE4A (A), PDE4B (B) and PDE4D (C). Significant reduction of PDE4D isoforms PDE4D3 (D) and PDE4D9 (E) following miR-208a expression. No significant effect of miR-208a on PDE1A (F), PDE3A (G) and PDE8B (I) with the exception PDE8A which was increased (H). Data were analyzed by t-test and expressed as mean ± SEM, n=3 independent experiments, P <0.05.

**Figure S4. Suppression of PDE4D by miR-208a.**

Representative immunohistochemistry images are shown for untreated (A), miR-208a mutant (B) and miR-208a (C) treated myocytes. To help visualize myocytes in miR-208a treated cells DAPI stained miR-208a treated myocytes are shown in (D). The images were taken with 20x objective Zeiss confocal microscopy. Scale bar = 100µm.

**Figure S5. Time course of suppression of PDE4D post miR-208a gene delivery.**

Representative immunohistochemistry images are shown for day 1 untreated (A), miR-208a mutant (B) and miR-208a (C); and for day 2 untreated (D), miR-208a mutant (E) and miR-208a (F); and for day 3 untreated (G), miR-208a mutant (H) and miR-208a (I). To help visualize the presence of myocytes, we additionally did immunohistochemistry staining for sarcomeric actinin that was counterstained with DAPI marking the nucleus of myocytes. The images were taken with 20x objective Zeiss confocal microscopy. Scale bar = 100µm.

**Figure S6. Absence of an effect of miR-208a on PDE5A expression.**

Expression of control PDE5A as assessed by immunohistochemistry (A-C). No significant reduction of PDE5A staining was observed using a PDE5A specific antibody in miR-208a transduced and in control myocytes (A-C). The images were taken with 40x objective confocal microscopy and bar shows scale of 50µm. A similar result was observed by Western blot (D, E). The original Western blots for these figures are shown in Figure S16.

**Figure S7.**  **Experimental validation of 3´UTR of PDE4D as a miR-208a target site.**

Panel (A) shows schematic of the construct used to test the function of miR-208a where 3´UTR of PDE4D is linked to downstream of the GFP cDNA. Panel (B) shows Western blot analysis of suppression of GFP expression in HEK cells after co-transfection of miR-208a and 3´UTR of PDE4D linked to downstream of GFP cDNA. Accordingly, the GFP protein was reduced by miR-208a only in HEK cells that contained PDE4D 3’UTR (lanes 5, 7). The administration of miR-208a antagomir counteracts the activity of miR208a resulting in no GFP suppression (lane 8). The use of scrambled antagomir did not block activity of miR-208a (lane 7). The original Western blots for these figures are shown in Figure S17. Summary of relative expression of the GFP obtained from Western blot analysis shown in (C) after normalization against GFP alone (lane1), P<0.05. Actin was used as loading control and for normalization of GFP protein in each sample. Data are shown as mean +/- SEM, n= 3; *P<0.05.

**Figure S8. Suppression of PRKAR1α by miR-208a.**

Two sites of PRKAR1α 3" UTR sequence alignment across species highlighting the seed match sequences (red) that is complementary for the seed sequences of miR-208a (A). The mismatches are shown in black letters, and 3’compensatory sites are in light blue. The PRKAR1α content for adult myocytes (B,C) and miR-208a transgenic mice (D,E) are shown with representative Western blots and summary plots. The original Western blots for these figures are shown in Figure S18. The cropped regions of the Western blots used in the manuscript are underlined in the original Western blot. Lanes in the western blot labeled with letter A are for other experiments not used in this paper. Data are shown as the means +/- SEM. N=4 mice * P < 0.05 by one-way ANOVA.

**Figure S9. Experimental validation of 3´UTR of THRAP1 as a miR-208a target.**

Panel (A) shows schematic of the construct used to test the function of miR-208a where 3´UTR of THRAP1 is linked to downstream of the GFP cDNA. Representative live images are shown for ventricular myocytes transduced with AdGFP-THRAP13’UTR (B), admiR-208a and AdGFP-THRAP13’UTR at 1:10 dilution (C) and AdmiR-208a and AdGFP-THRAP13’UTR at 1: 10^5^ dilution (D) treated myocytes. The images were taken with 5x objective AxioVision microscopy. Scale bar = 200µm.

**Figure S10. Increased expression of cardiac expressed miRs targeting PDE4D in the miR-208a expressing heart.**

Hearts from miR-208a Tg animals have increased expression of several miRs targeting PDE4D, including miR-101 (A), miR-30b (B), miR-26b (C) and miR-18a (D). For adenoviral mediated miR-208a expression in adult ventricular rat myocytes, only miR-101 increased and the rest miRs are not statistically different (E-H).
