# Supplementary material for: Molecular inotropy mediated by cardiac miR-based PDE4D/PRKAR1α/phosphoprotein signaling
Source: Sci Rep. 2016 Nov 11;6:36803. doi: 10.1038/srep36803 (PMC5105063; doi:10.1038/srep36803)

**Molecular inotropy mediated by cardiac miR-based**

**PDE4D/PRKAR1α/phosphoprotein signaling**

Fikru B. Bedada^1^, Joshua J. Martindale^1^, Erik Arden^1^ and Joseph M. Metzger^1^

^1^Department of Integrative Biology and Physiology, University of Minnesota Medical School, 6-125 Jackson Hall, 321 Church Street SE, Minneapolis, MN 55455 U.S.A
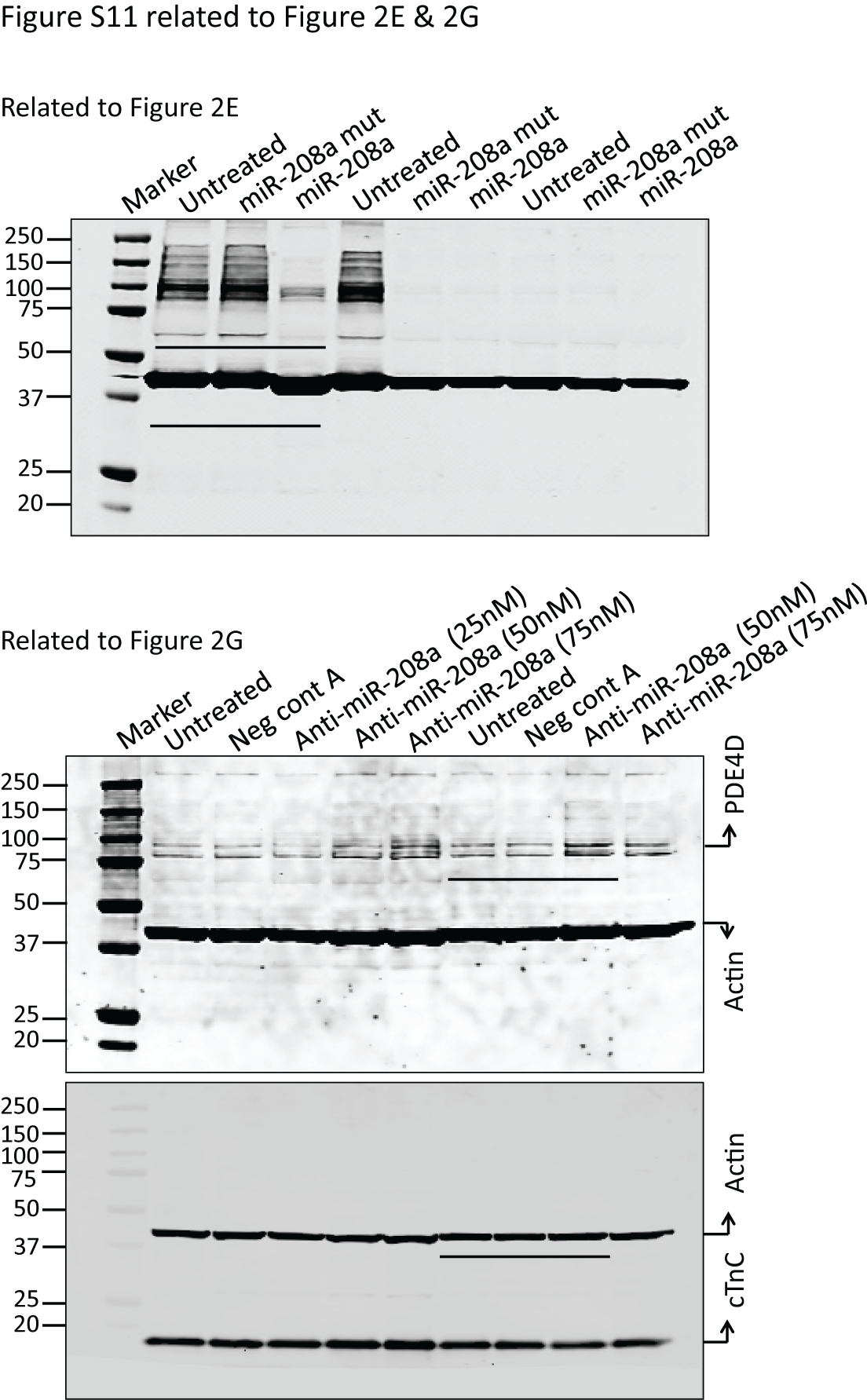

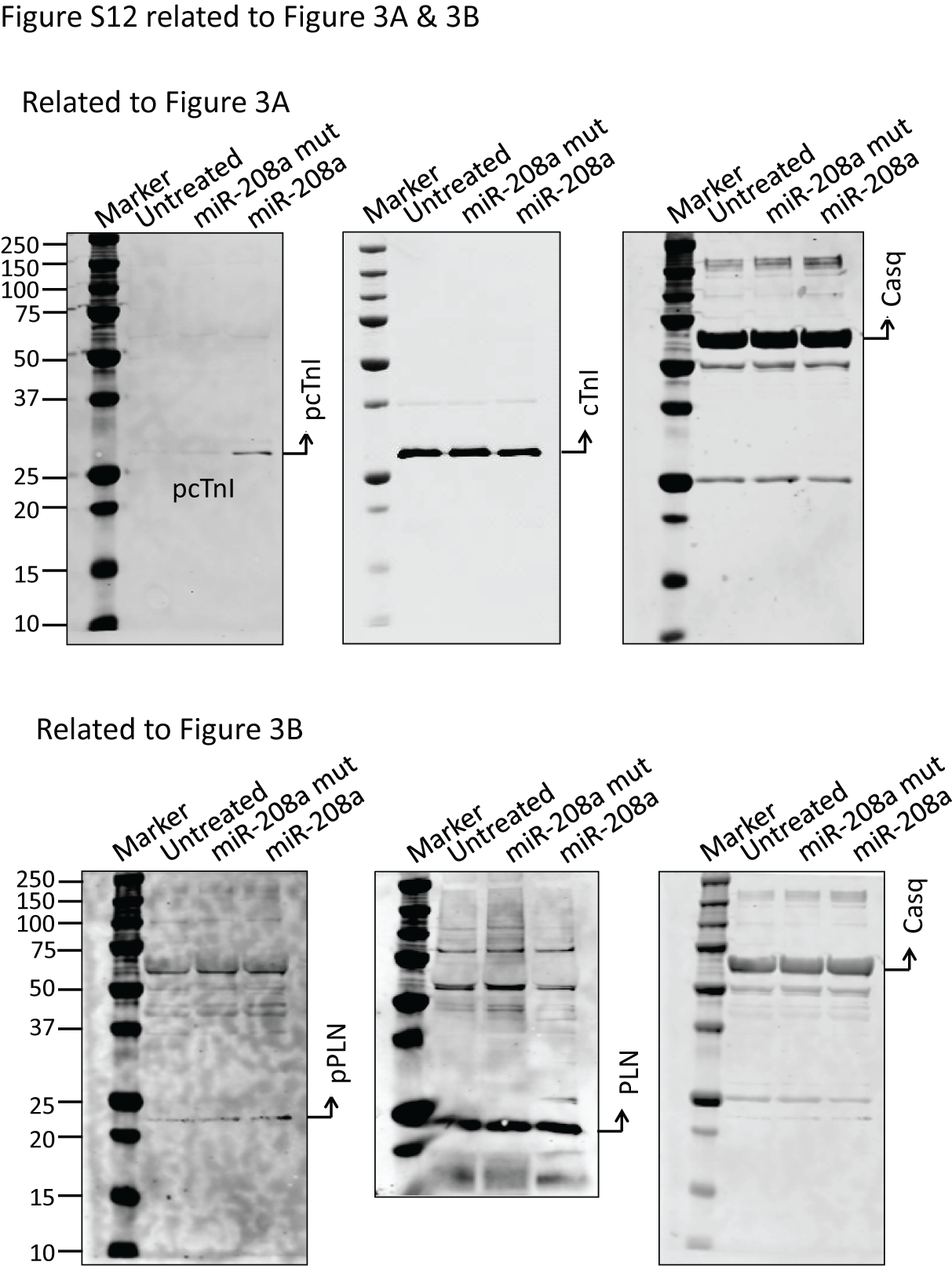

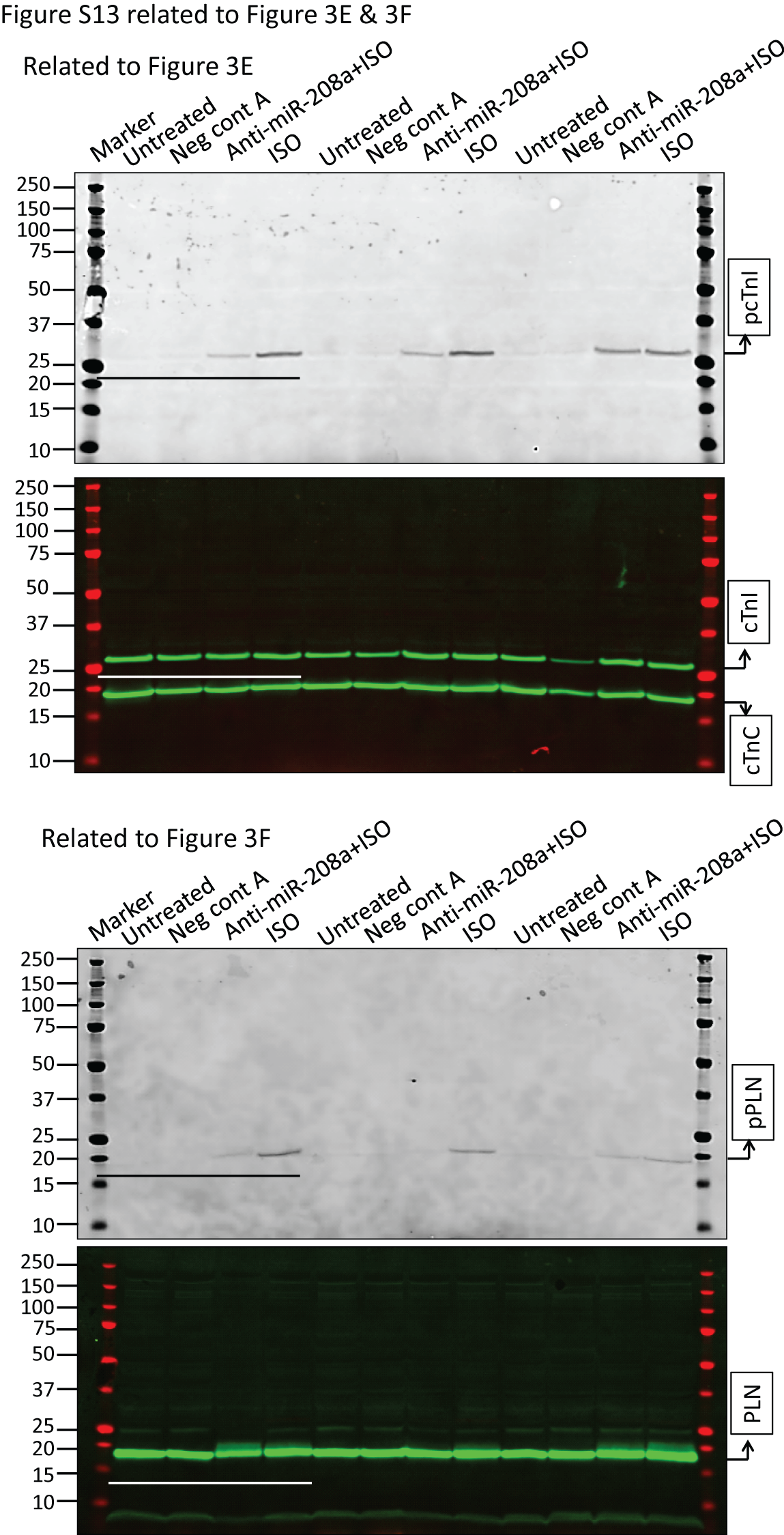

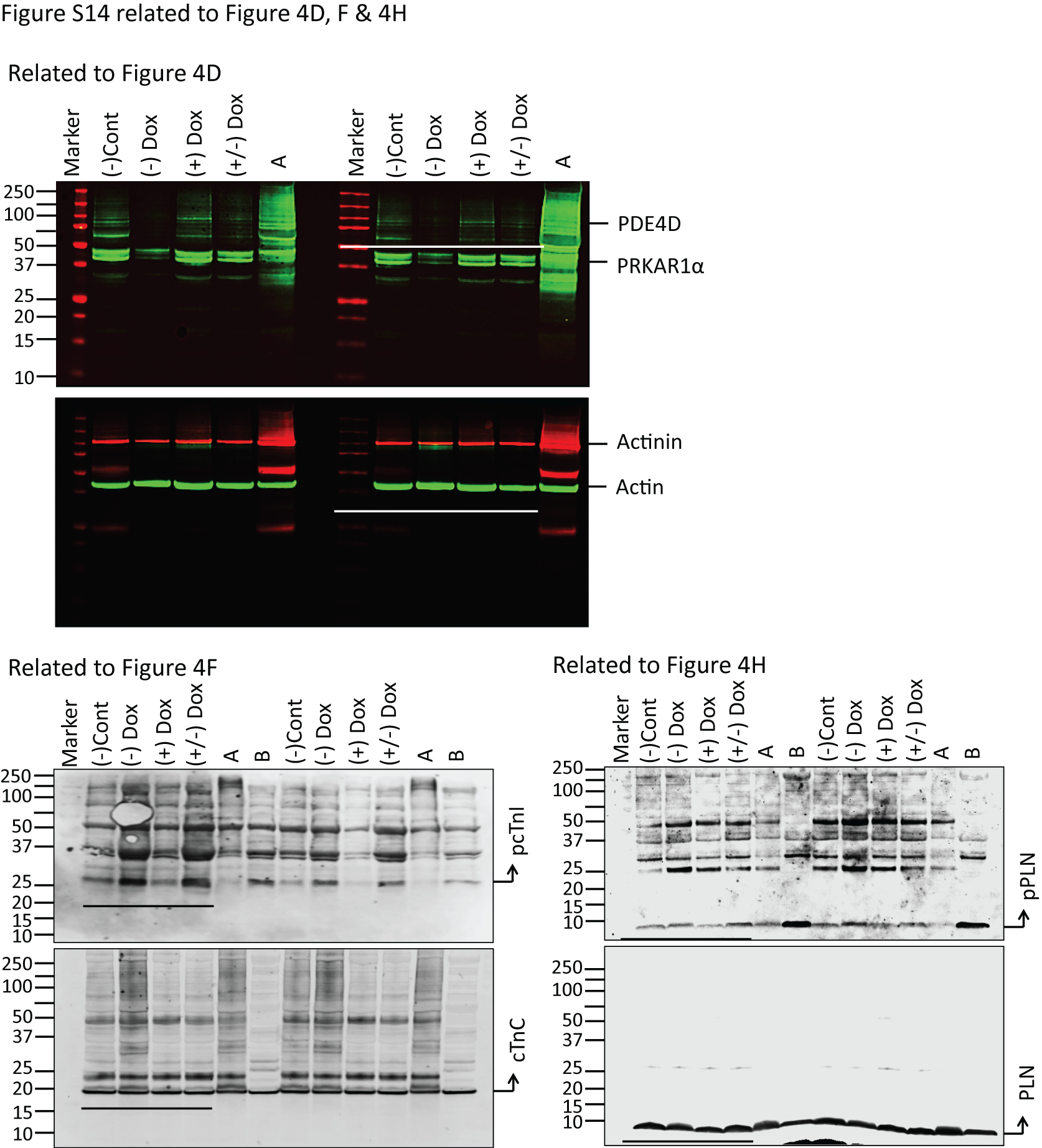

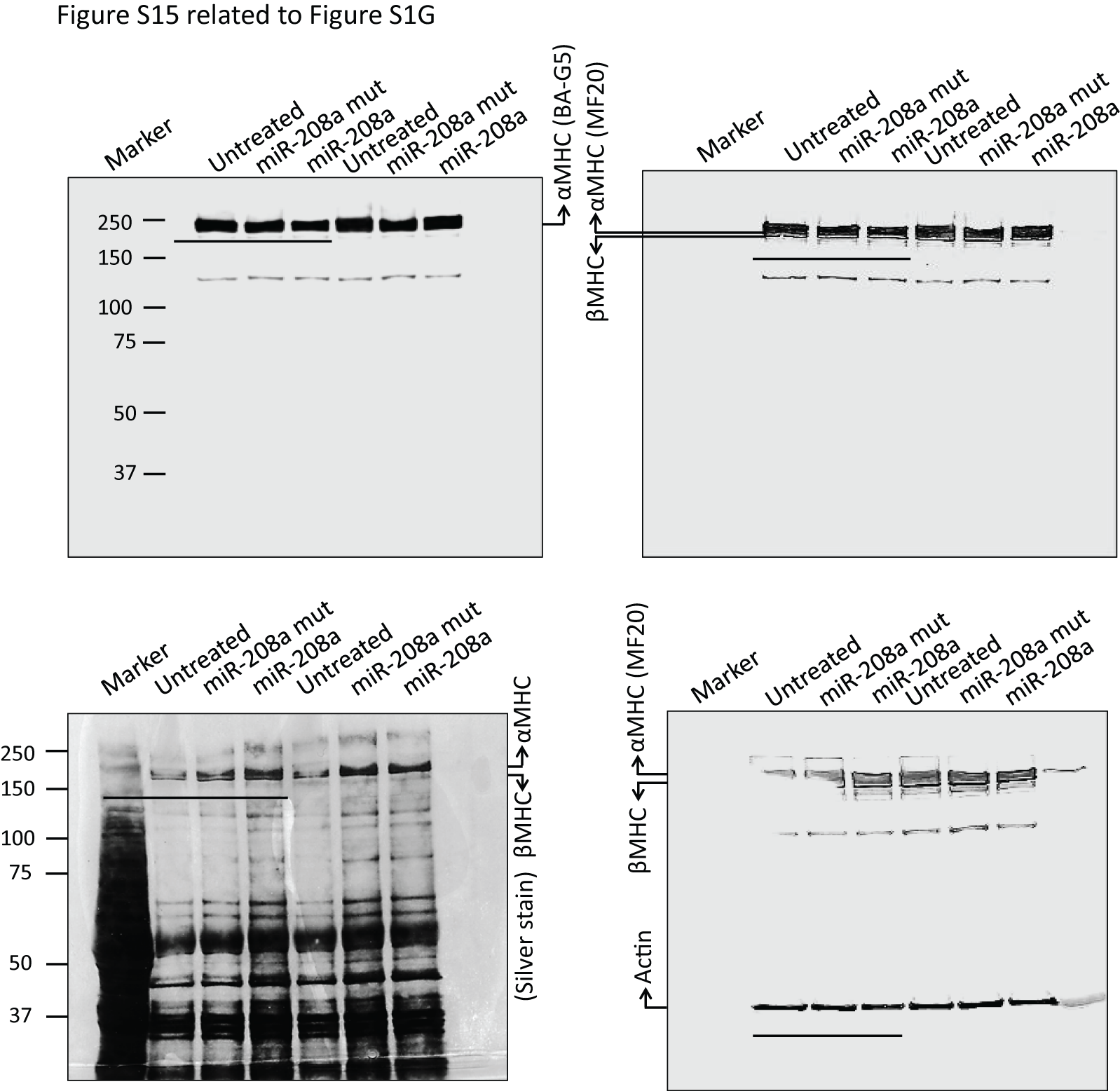

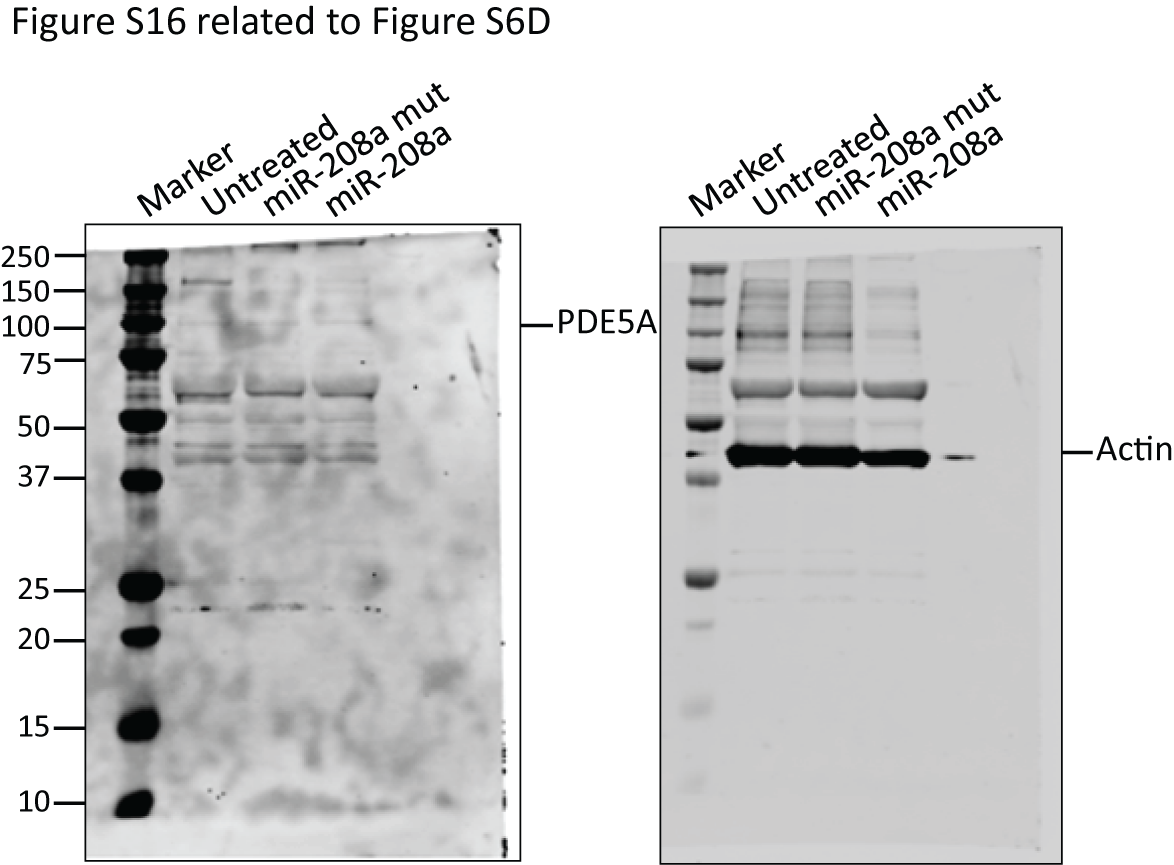

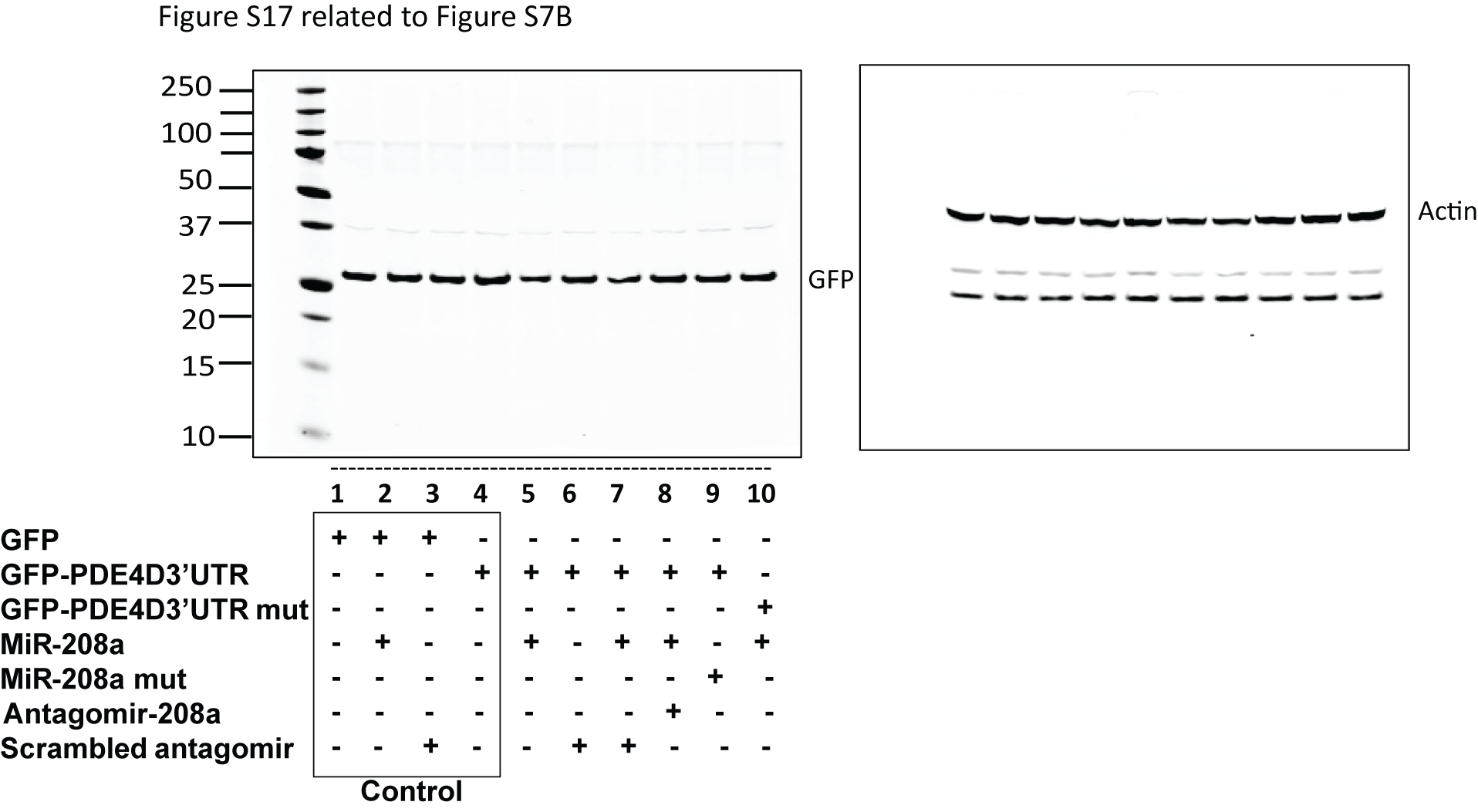

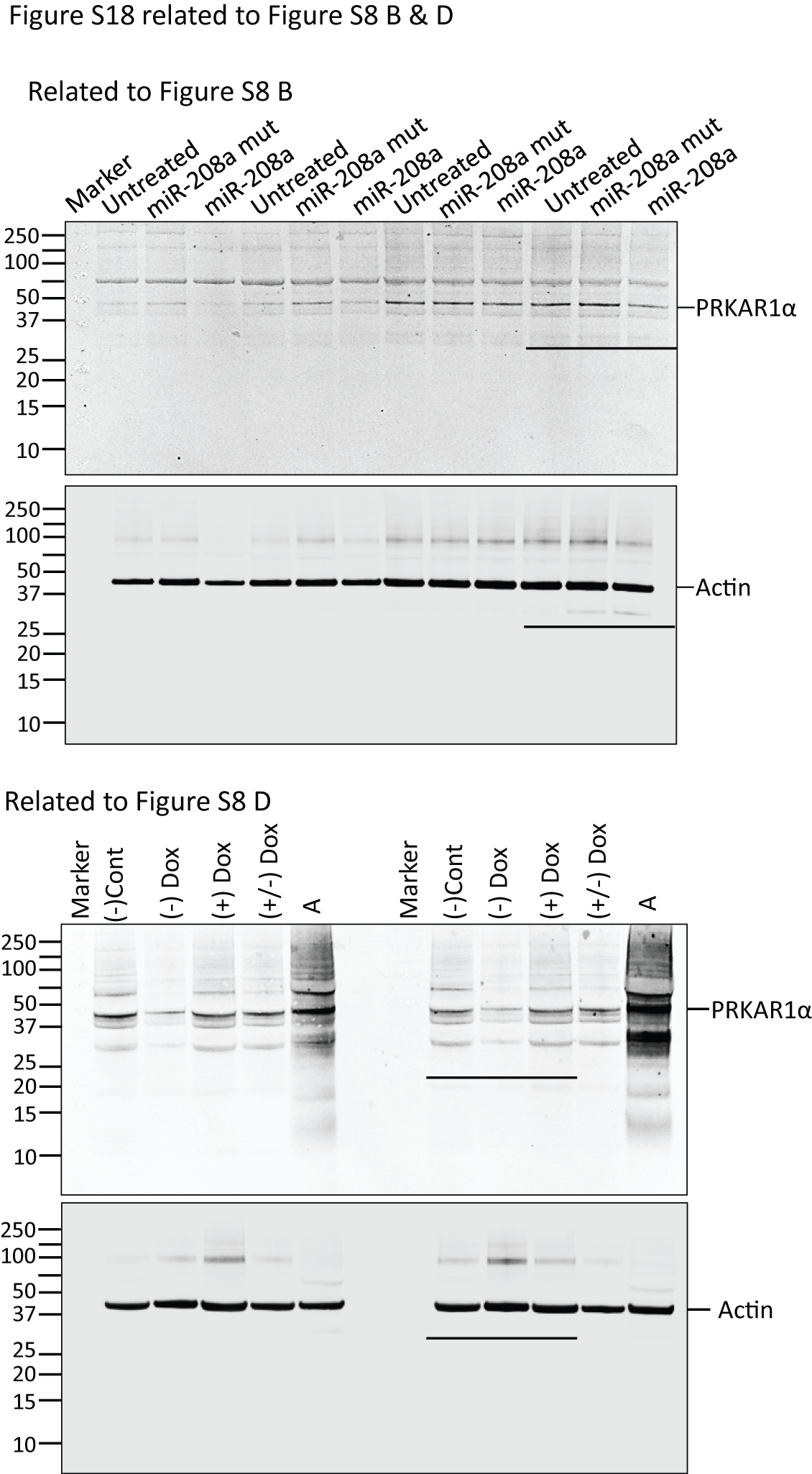

Supplement: Supplementary Figures [file srep36803-s2.doc]
